# Manifold-constrained nucleus-level denoising diffusion model for structure-based drug design

**DOI:** 10.1073/pnas.2415666122

**Published:** 2025-10-06

**Authors:** Shengchao Liu, Liang Yan, Weitao Du, Weiyang Liu, Zhuoxinran Li, Hongyu Guo, Christian Borgs, Jennifer Chayes, Anima Anandkumar

**Affiliations:** ^a^Department of Electrical Engineering and Computer Sciences (EECS), University of California, Berkeley, CA 94720; ^b^Department of Computing and Mathematical Sciences (CMS), California Institute of Technology, Pasadena, CA 91125; ^c^Institute of Science and Technology for Brain-Inspired Intelligence (ISTBI), Fudan University, Shanghai 200433, China; ^d^Alibaba DAMO Academy, Bellevue, WA 98004; ^e^Max Planck Institute for Intelligent Systems, Tübingen 72076, Germany; ^f^Department of Human Geography, University of Toronto, Toronto, ON M5S 1A1, Canada; ^g^National Research Council Canada, Ottawa, ON K1A 0R6, Canada

**Keywords:** structure-based drug design, statistical machine learning, manifold learning, generative AI

## Abstract

AI models have shown great potential in structure-based drug design, generating ligands with high binding affinities. However, existing models have often overlooked a crucial physical prior: Atoms must maintain a minimum pairwise distance to avoid atomic collision, a phenomenon governed by the balance of attractive and repulsive forces. To mitigate such atomic collisions, we propose NucleusDiff. It enforces spatial distance constraints between atomic nuclei and auxiliary mesh points placed on a spherical surface around each atom, approximating van der Waals boundaries to reduce atomic collisions. We quantitatively evaluate NucleusDiff using the CrossDocked2020 dataset and a COVID-19 therapeutic target, demonstrating that NucleusDiff reduces collision rate by up to 100.00% and enhances binding affinity by up to 22.16%, surpassing state-of-the-art models for structure-based drug design. We also provide qualitative analysis through manifold sampling, visually confirming the effectiveness of NucleusDiff in reducing atomic collisions and improving binding affinities.

## Significance

Structure-based drug design is a critical field in chemistry and biology, and recent advancements in machine learning have significantly enhanced the generation of ligands with high binding affinities for target proteins. However, existing machine learning methods have been overlooking a crucial physical prior: Atoms must maintain a minimum pairwise distance to avoid atomic collisions. We address this issue by introducing a learning approach that incorporates motivated geometric constraints to improve the physical plausibility and binding affinity of generated molecular structures. Our results demonstrate improved molecular realism and practical benefits for drug design tasks.

Structure-based drug design is a cornerstone of drug discovery, aiming at designing small molecule ligands based on the geometric structures of biological targets, typically the protein pockets. It faces significant challenges due to the vast chemical search space and the complex geometric interactions between ligands and proteins in three-dimensional Euclidean space (1). To cope with these challenges, machine learning has emerged as a powerful tool for efficiently navigating the design space of small molecules and effectively generating ligands with high binding affinities.

**Fig. 1.**
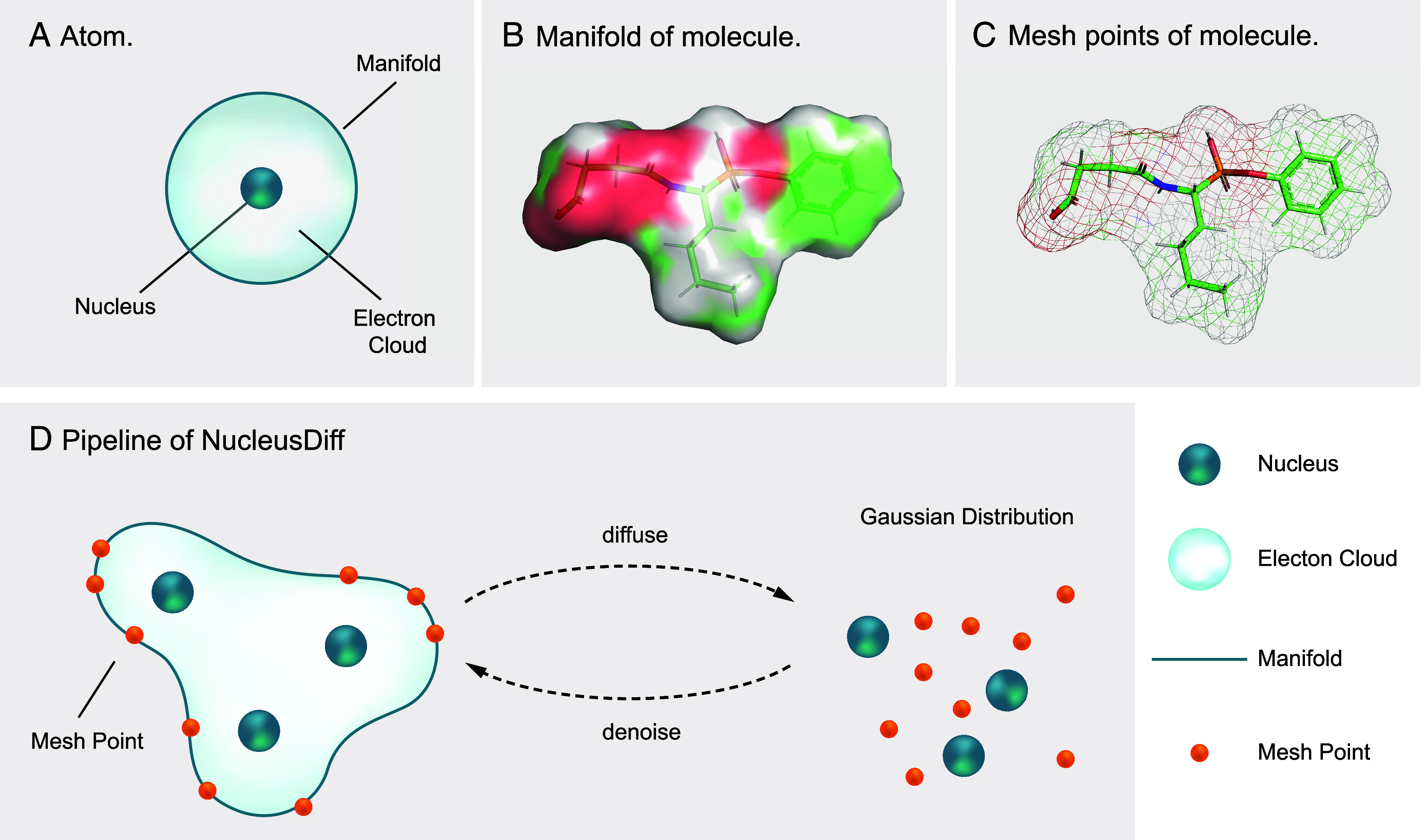
(*A*) Illustration of the atomic nucleus and the geometric manifold of an atom. The manifold represents the spatial boundary defined by the van der Waals radius, which sets the minimum distance between atomic nuclei. (*B*) Illustration of the manifold surrounding a molecule. (*C*) Illustration of the mesh points obtained from discretizing a manifold. (*D*) Pipeline of NucleusDiff. NucleusDiff performs denoising diffusion on both the nuclei and the discretized mesh points, where the distances between them approximate the van der Waals radii.

**Fig. 2.**
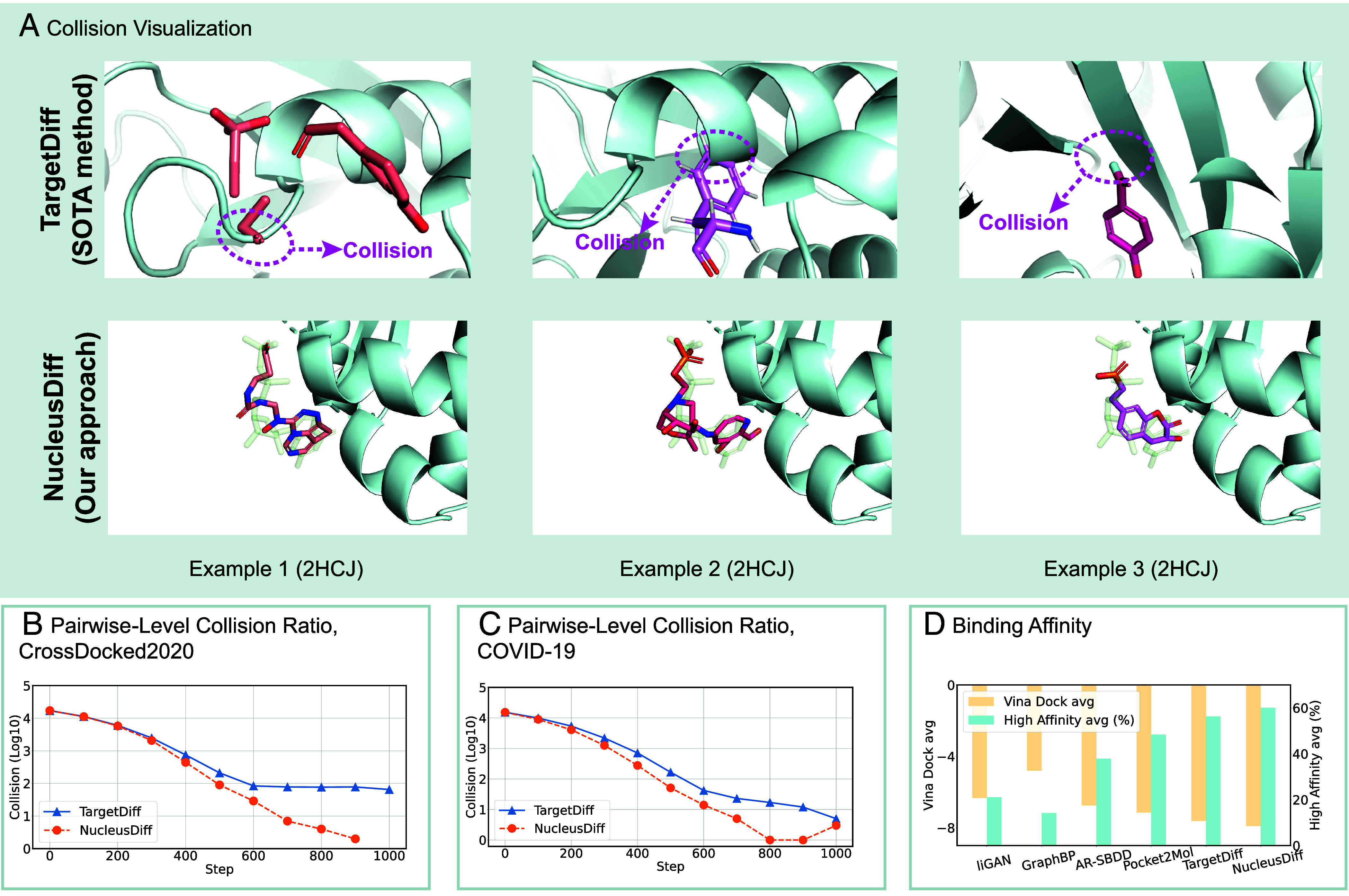
(*A*) Visualization of generated ligands for the target 2HCJ. (*B*) Visualization of the pairwise-level collision ratio in TargetDiff and NucleusDiff during the inference on the CrossDocked2020 dataset. (*C*) Visualization of the pairwise-level collision ratio in TargetDiff and NucleusDiff during the inference on the COVID-19 therapeutic target. (*D*) Visualization of the binding affinities (in Vina Dock) for 10 K sampled ligands and given proteins.

State-of-the-art deep generative models for the task generate ligands with high binding affinities by leveraging physical properties, such as the equivariance of molecular systems to rotations and translations (2–8). However, they suffer from certain limitations due to the approximations in modeling. For instance, they treat each atom as a solid point, which does not fully reflect the spatial extent that real atoms occupy in three-dimensional space. Instead of being single points, atoms occupy a finite spatial region around each nucleus due to the distribution of their electrons, which leads to an effective minimum distance between atoms in a molecule. As illustrated in Fig. 1*A*, these factors collectively influence the distribution and arrangement of electrons, adhering to a physical prior that requires atoms to maintain a minimum pairwise distance to avoid atomic collision. Having overlooked this principle, current deep generative models could breach the constraints and result in atomic collision, where two atoms in the generated ligand–pocket pairs are positioned too close to each other, as depicted in Fig. 2.

One straightforward approach to address this issue is to consider constraining atomic pairwise distance into deep learning models as a regularization term. However, since this is an atom-pairwise measure, the computational complexity scales quadratically with the number of atoms. Therefore, a more efficient and effective method is necessary to address the atomic collision problem.

## 1.1. Our Contributions.

To tackle this challenge, we propose NucleusDiff. NucleusDiff operates in two phases. The first phase, illustrated in Fig. 1*B*, incorporates inductive biases based on established physical priors: NucleusDiff uses a geometric manifold, which approximates the spatial boundary around each atomic nucleus defined by its van der Waals radius. In the second phase, shown in Fig. 1*C*, NucleusDiff discretizes the manifold into a set of triangle mesh points. Then, based on this, NucleusDiff uses a regularization term to align the distance between nuclei and sampled mesh points with the van der Waals radii. This alignment implicitly maintains proper pairwise atomic distances, and the complexity of modeling such a constraint increases linearly with the number of atoms, making NucleusDiff an efficient approach.

We verify the effectiveness of NucleusDiff using 100 K protein–ligand binding complexes from the CrossDocked2020 (9) first. Our quantitative analysis demonstrates that NucleusDiff significantly outperforms the state-of-the-art models. NucleusDiff demonstrates promising performance by achieving a 22.16% improvement in binding affinity (Vina Score) compared to the previous state-of-the-art method [TargetDiff (7)] on CrossDocked2020. Owing to our design, NucleusDiff effectively minimizes atomic collisions, achieving an almost negligible collision rate, with reductions reaching up to 100.00%. Additionally, our case study on the COVID-19 therapeutic target shows that NucleusDiff not only achieves a superior binding affinity, with an improvement of up to 21.37% but also reduces the collision rate by up to 66.67%.

In summary, the proposed NucleusDiff reaches an optimal balance in maintaining proper physical distances to avoid atomic collision and preserving the critical biochemical properties including binding affinity and stability. We believe this work demonstrates how integrating motivated geometric constraints into generative models can enhance the design of biologically relevant molecules for structure-based drug discovery, offering a practical approach for improving molecular generation in this field.

## 2. Results

### 2.1. Background.

#### 2.1.1. Small molecules and proteins.

In this work, we consider small molecule ligands, which are sets of atoms in the 3D Euclidean space, $\left\{v^{L},x^{L}\right\}$, where $v^{L}$ represents the atomic number and $x^{L}$ represents the atomic nucleus coordinate. Proteins are macromolecules, *i.e.*, chains of residues. In nature, there are 20 types of residues. Each residue is a small molecule, with a fixed backbone structure: a basic amino group, an acidic carboxyl group, a side chain that is unique to each amino acid, and a carbon $C_{\alpha }$ connecting three components. In this work, we consider modeling proteins in the backbone-level information, *i.e.*, the backbone atomic number and backbone atomic nucleus coordinates, $\left\{v^{P},x^{P}\right\}$.

#### 2.1.2. Nucleus and manifold.

Each atom is modeled as a nucleus with a surrounding spatial boundary, characterized by its van der Waals radius, as illustrated in Fig. 1. Recent works have employed manifold learning over such spatial boundaries for molecule property prediction (10, 11) and protein modeling in structure-based drug design (12). In our approach, we model a geometric manifold around each atomic nucleus in the ligand, where the radius is set to the van der Waals value. This manifold, sometimes referred to as the van der Waals surface or the solvent-accessible surface (10), provides a natural geometric constraint for molecular generation. Then we discretize the manifold into triangle mesh points, a form suitable for computational analysis. This is implemented using the Python package PyMesh (13). For notation, for each ligand atom $\left(v^{L},x^{L}\right)$, the coordinate and type of nuclei are the same as the atom-level, *i.e.*, $v^{N}≜v^{L}$ and $x^{N}≜x^{L}$. The coordinate of the discretized points on the manifold is marked as $x^{M}$. Notice that we use a special token to delegate the electron points on the manifold.

#### 2.1.3. Structure-based drug design.

The structure-based drug design (SBDD) task utilizes the geometric structures of proteins to design and optimize ligands, like small molecules. This can be formulated as a conditional distribution modeling problem, $p(x^{L},v^{L}|v^{P},x^{P})$. Notice that NucleusDiff improves this objective function by introducing nucleus-level modeling combined with manifold-sensitive constraints of small molecules, and the problem formulation becomes $p(x^{N},v^{N},x^{M}|v^{P},x^{P})$. More details will be discussed in *Materials and Methods*.

### 2.2. Atomic Collision.

and Collision Ratio Metrics The atomic collision occurs when two atoms come too close to each other, contradicting the physical constraints. We introduce the covalent radius *d* to measure it, as the covalent radius is a more strict quantity involving the covalent bonding. Suppose we have one ligand atom coordinate $x_{i}$, one protein atom coordinate $x_{j}$, and the corresponding covalent radii are $d_{i}$ and $d_{j}$, respectively. Then during the sampling stage for ligand generation, if two atoms get too close to each other, *i.e.*, $\|x_{j}-x_{j}\|\leq D_{\mathrm{ij}}=d_{i}+d_{j}$, we refer to this as the atomic collision problem. To quantify this in existing deep generative models, we propose three ratio metrics from three granularities. For clarity, we only present one of the representative metrics in the main article.

#### 2.2.1. Pairwise-level collision ratio (PLCR).

The metric is the atom PLCR, which quantifies the collision ratio between all the ligand atoms and protein atoms. For each ligand atom ($x_{i}^{L}$), we extract its *K* nearest protein atoms within the binding site. Then the PLCR is defined as

[1] $$\begin{array}{c} \begin{array}{c} \text{PLCR}=\frac{\sum_{k\in N_{\text{mol}},i\in N_{\text{atom}}^{k},j\in N_{\text{nearest}}^{i}}1(\|x_{i}-x_{j}\left\|\;<\;D_{\mathrm{ij}}\right)}{K\;\cdot \;\sum_{k\in N_{\text{mol}}}N_{\text{atom}}^{k}}, \end{array} \end{array}$$

where $N_{\text{mol}}$ is the number of ligand molecules, $N_{\text{atom}}^{k}$ is the number of atoms in the *k*-th molecule, $N_{\text{nearest}}^{i}$ is the number of the nearest protein atoms of the *t*-th ligand atom, and 1(·) is the indicator function.

We present the other two metrics in *SI Appendix*. The proposed collision ratio metrics assess the atomic collisions between pockets and generated ligands, enhancing our understanding of the generative model’s inference process for structure-based drug design. We initially test them on the generated ligands by a state-of-the-art generative model (7). As illustrated in Fig. 2, existing works exhibit the atomic collision issue to a certain extent.

To overcome this collision, these metrics can be directly incorporated into the training objective when modeling, but the computational complexity of these metrics is as high as $O(N_{\mathrm{mol}}N_{\mathrm{atom}}^{2})$, where $N_{\mathrm{mol}}$ is the number of molecules and $N_{\mathrm{atom}}$ is the combined number of atoms in the molecule and the protein pocket. Subsequently, we introduce NucleusDiff, an efficient model with higher binding affinities and lower atomic collisions.

### 2.3. Manifold-Constrained Nucleus-Level DDPM: NucleusDiff.

We propose NucleusDiff to reduce the atomic collision in the deep generative model for the structure-based drug design. The main idea is to jointly model the atomic nucleus and the mesh points of the surrounding manifold using a denoising diffusion model. In this section, we provide a brief introduction to NucleusDiff, and more detailed descriptions can be found in *Materials and Methods*.

#### 2.3.1. Diffusion model for geometry generation.

We first introduce the denoising diffusion model for density estimation on general geometries, x. The denoising diffusion probabilistic model (DDPM) consists of two stages: a forward and a backward process (14). The forward process gradually adds noise to the input geometric data $x_{0}=x$ to a prior Gaussian distribution $x_{T}$, and the backward process is the denoising process from the prior distribution to the data distribution. In concrete, suppose the data distribution is x∼q(x), and we have *T* forward and backward steps with the scheduled variance ${\left\{\beta_{t}\right\}}_{t=1}^{T}$. Then each forward step can be represented as $q\left(x_{t}|x_{t-1}\right)=N\left(x_{t};\;\sqrt{1-\beta_{t}}x_{t-1},\beta_{t}I\right)$, which gives $q\left(x_{t}|x_{0}\right)=N\left(x_{t};\;\sqrt{{\overset{¯}{\alpha }}_{t}}x_{0},\left(1-{\overset{¯}{\alpha }}_{t}\right)I\right)$, where $\alpha_{t}=1-\beta_{t}$ and ${\overset{¯}{\alpha }}_{t}=\prod_{s=1}^{t}\alpha_{s}$. Following the Bayes theorem, the posterior $q(x_{t-1}|x_{t},x_{0})$ can also be expressed as a Gaussian distribution:

[2] $$\begin{array}{c} q\left(x_{t-1}|x_{t},x_{0}\right)=N\left(x_{t-1};\;\tilde{\mu }\left(x_{t},x_{0}\right),{\tilde{\beta }}_{t}I\right), \end{array}$$

where $\tilde{\mu }\left(x_{t},x_{0}\right)=\frac{\sqrt{{\overset{¯}{\alpha }}_{t-1}}\beta_{t}}{1-{\overset{¯}{\alpha }}_{t}}x_{0}+\frac{\sqrt{\alpha_{t}}(1-{\overset{¯}{\alpha }}_{t-1}}{1-{\overset{¯}{\alpha }}_{t}}x_{t}$ and ${\tilde{\beta }}_{t}=\frac{1-{\overset{¯}{\alpha }}_{t-1}}{1-{\overset{¯}{\alpha }}_{t}}$. The goal is to maximize the log-likelihood of data distribution p(x), and after reparameterization, we aim to directly predict the ground-truth coordinates $x_{0}$ with a parameterized network ${\hat{x}}_{0}=\phi_{\theta }\left(x_{t},t\right)$. The training objective is

[3] $$\begin{array}{c} \begin{array}{c} L_{t-1}\left(x\right)=E_{q}[{\left\|x_{0}-{\hat{x}}_{0}\right\|}^{2}]. \end{array} \end{array}$$

Please refer to the DDPM paper for detailed derivations (14).

Eq. **3** holds for arbitrary density estimation, and in the following paragraphs, we will discuss how we adapt this for our proposed NucleusDiff for structure-based drug design.

#### 2.3.2. Nucleus-level diffusion model for ligand generation.

In our task, the goal is to model the atomic types and coordinates in ligands given the pocket structure: $p(v^{L},x^{L}|v^{P},x^{P})$. The training objective includes a categorical term $L_{t-1}\left(v^{L}\right)$ on atomic types and a continuous term $L_{t-1}\left(x^{L}\right)$ on atomic coordinates (7). Recall that with loss of generality, we can interchange atom information (type and coordinate) with nucleus information (type and coordinate), *i.e.*, $v^{N}=v^{L}$ and $x^{N}=x^{L}$.

In this paragraph, we mainly discuss the continuous objective $L_{t-1}\left(x^{N}\right)$, while the discrete objective function $L_{t-1}\left(v^{N}\right)$ over atomic type is described in *Materials and Methods*. Then adopting Eq. **3** for atomic coordinates, the training objective becomes

[4] $$\begin{array}{c} \begin{array}{cc} L_{t-1}\left(x^{N}\right) & =E_{q}[{\left\|x_{0}^{N}-{\hat{x}}_{0}^{N}\left(x_{t}^{N},t,v^{P},x^{P}\right)\right\|}^{2}]. \end{array} \end{array}$$

We note that the parameterized network ${\hat{x}}_{0}^{N}$ should be equivariant to the rotations and translations of the whole molecular system (14–16). On the other hand, recent works (8) claim that such SE(3)-equivariant modeling is unnecessary for achieving high binding affinities in structure-based drug design tasks. Meanwhile, the manifold constraint introduced in NucleusDiff is agnostic to the design of the parameterized network, and we follow the state-of-the-art method in this work (7).

#### 2.3.3. Manifold-constrained nucleus-level diffusion model for ligand generation.

To reduce the atomic collision, we introduce an extra constraint term that keeps the distance between the nucleus and the mesh points over the manifold as van der Waals radius, *R*. This constraint ensures that the atomic distances are maintained within reasonable bounds, preventing geometric overlaps. To adopt this into modeling, for each nucleus, we obtain its *K* closest mesh points in the manifold, marked as $x^{M}$. Thus, the goal becomes to maximize the joint distribution of nuclei and mesh points conditioned on the pocket, as $p(v^{N},x^{N},x^{M}|v^{P},x^{P})$. To adapt this into Eq. **3**, the objective of the sampled mesh points is

[5] $$\begin{array}{c} \begin{array}{cc} L_{t-1}\left(x^{M}\right) & =E_{q}[{\left\|x_{0}^{M}-{\hat{x}}_{0}^{M}\left(x_{t}^{M},t,v^{P},x^{P}\right)\right\|}^{2}]. \end{array} \end{array}$$

On the other hand, recall that the mesh points are scattered around the nuclei with a fixed van der Waals radius *R*. Thus we add a regularization term by forcing the distance between each corresponding mesh point $x_{j}^{N}$ and nucleus $x_{i}^{M}$ to be close to *R*:

[6] $$\begin{array}{c} \begin{array}{cc} L_{t-1}\left(x^{N},x^{M},R\right) & =\sum_{i}\sum_{j}\left\|\right\|{\hat{x}}_{0}^{N}\left(x_{t}^{N},t,v^{P},x^{P}\right)\\ & -{\hat{x}}_{0}^{M}\left(x_{t}^{M},t,v^{P},x^{P}\right)\left\|-R_{\mathrm{ij}}\right\|. \end{array} \end{array}$$

To be precise, we adopt a slight decoupling trick in implementing the regularization term in Eq. **6**. That is, the denoised outputs ${\hat{x}}_{0}^{N}$ and ${\hat{x}}_{0}^{M}$ are not necessarily derived from the same timestep *t*, but rather from potentially different timesteps $t_{N}$ and $t_{M}$ for the nucleus and mesh points, respectively. This design choice improves training flexibility and stability, allowing the network to independently refine the representations of atomic centers and manifold surfaces.

[7] $$\begin{array}{c} \begin{array}{cc} L_{\text{reg}}\left(x^{N},x^{M},R\right) & =\sum_{i}\sum_{j}\left\|\right\|{\hat{x}}_{0}^{N}\left(x_{t_{n}}^{N},t_{n},v^{P},x^{P}\right)\\ & -{\hat{x}}_{0}^{M}\left(x_{t_{m}}^{M},t_{m},v^{P},x^{P}\right)\left\|-R_{\mathrm{ij}}\right\|. \end{array} \end{array}$$

We call such a manifold-constrained nucleus-level modeling as NucleusDiff. The overall objective function of the NucleusDiff is

[8] $$\begin{array}{c} \begin{array}{cc} L & =E_{t_{n}}\left[L_{t_{n}-1}\left(x^{N}\right)+L_{t_{n}-1}\left(v^{N}\right)\right]\\ & +E_{t_{m}}\left[L_{t_{m}-1}\left(x^{M}\right)\right]+E_{t_{n},t_{m}}\left[L_{t-1}\left(x^{N},x^{M},R\right)\right]. \end{array} \end{array}$$

### 2.4. Experimental Setup.

#### 2.4.1. Datasets.

We conduct experiments on two datasets. 1) We first utilize CrossDocked2020 (9) to train and evaluate our model. Following the approach of ref. 4, we refine the dataset of 22.5 million docked protein–ligand binding complexes by selecting the poses with a rms deviation (RMSD) of less than 1 Å and protein sequence identity below 30%. Ultimately, we have 100,000 complexes for training and 100 complexes for testing. 2) We also carry out experiments on the COVID-19 target. Additionally, we assess the generalization abilities of NucleusDiff on two other real-world therapeutic targets, as detailed in *SI Appendix*.

#### 2.4.2. Mesh point construction.

We utilize MSMS (17) to compute the solvent-excluded surface of the molecule. It generates a triangular mesh data structure for small molecules, and we set the argument probe radius to 1.5 Å and sampling density argument to 3.0^*^. MSMS possesses certain challenges such as degenerate vertices and disconnected surfaces, which can disrupt the uniform distribution of mesh points. We employ PyMesh to overcome these issues (13). It enhances the mesh quality by reducing the vertex count and correcting errors in poorly meshed regions. Finally, we select the *K* mesh points that are closest to the van der Waals radius from the nucleus to construct a mesh point dataset. Notably, this mesh point data structure, which predominantly includes the 3D coordinates of the mesh points, is only required during the learning phase and not during inference.

#### 2.4.3. Baselines.

For benchmarking, we compare with various baselines: liGAN (18), AR (4), Pocket2Mol (6), GraphBP (5), PMDM (19), and TargetDiff (7). liGAN (18) is a 3D CNN-based method that generates 3D voxelized molecular images following a conditional VAE scheme. AR (4), Pocket2Mol (6), and GraphBP (5) are GNN-based methods that generate 3D molecules by sequentially placing atoms into the protein binding pocket. We choose AR-SBDD (4) and Pocket2Mol (6) as representative baselines due to their outstanding performance. TargetDiff (7) and PMDM (19) are the state-of-the-art models in this research line, and they both employ a diffusion-based technique for generating atom coordinates and types.

### 2.5. Atomic Collision Evaluation on CrossDocked2020.

#### 2.5.1. The collision metrics.

To gain a comprehensive understanding of how the atomic collision problem evolves during the inference process of diffusion-based probabilistic models (14) for structure-based drug design, we compare NucleusDiff with the state-of-the-art model in this research line, TargetDiff (7). For both methods, we use 1,000 timesteps for training and inference. We analyze the PLCR metric for atomic collision at 11 timesteps, sampled every 100 intervals from Step-0 to Step-1000. We present the atomic collision from Step-700 to Step-1000 in Table 1, with the complete results available in *SI Appendix*.

**Table 1.** The performance among pocket–ligand pairs for structure-based drug design in the CrossDocked2020 and COVID-19 target

	CrossDocked2020		COVID-19 target	
Models	TargetDiff	NucleusDiff	TargetDiff	NucleusDiff
Step-700	78/2300930	7/2300930	23/210000	5/210000
Step-800	77/2300930	2/2300930	17/210000	1/210000
Step-900	78/2300930	4/2300930	12/210000	1/210000
Step-1000	65/2300930	0/2300930	05/210000	3/210000

#### 2.5.2. Analysis.

As shown in Table 1, TargetDiff maintains stable atomic collision ratios from Step-700 to Step-1000. In contrast, NucleusDiff demonstrates consistently lower atomic collision ratios during these inference steps. Notably, NucleusDiff outperforms TargetDiff by nearly an order of magnitude in terms of the PLCR metric. In the final sampling step, NucleusDiff achieves an almost negligible Collision ratio, further demonstrating its superior performance. As illustrated in Fig. 2, the convergence trends of NucleusDiff and TargetDiff differ markedly when evaluated using the PLCR metric, with NucleusDiff demonstrating a significantly more pronounced convergence. More comprehensive results and analyses of NucleusDiff’s atomic collision performance are provided in *SI Appendix*.

### 2.6. Binding Affinity Evaluation on CrossDocked2020.

#### 2.6.1. The general metrics.

The Vina Score, Vina Min, and Vina Dock metrics are employed to evaluate the binding affinity and potential biological efficacy of small molecule drug candidates in interaction with target proteins, via the computation of docking efficiency scores. Following the methodologies outlined in refs. 4, 6, and 7, we utilize the open-source AutoDockTools software (20) for these calculations. The High Affinity metric gauges the strength of the ligand-target protein interaction.

Additionally, we follow existing works and consider three more metrics (4, 6, 7). QED provides a numerical assessment of a compound’s drug-like characteristics, with higher values indicating a greater propensity for a compound to embody successful drug attributes. SA quantifies the ease with which a compound can be synthesized. Last, Diversity measures the range and heterogeneity of molecular structures and properties across a set of compounds.

#### 2.6.2. Analysis.

We generate 100 ligand molecules for each protein target in the test set, resulting in a total of 10,000 molecules. The size of each generated molecule, *i.e.*, the number of atoms in each molecule, is determined by sampling from the size distribution observed in the training set. The comprehensive results for NucleusDiff and the baselines are displayed in Table 2.

**Table 2.** A summary of 14 biochemical properties for reference ligands and molecules generated by baseline models and NucleusDiff

	Vina score (↓)		Vina min (↓)		Vina dock (↓)		High affinity (↑)		QED (↑)		SA (↑)		Diversity (↑)	
Metrics	Avg.	Med.	Avg.	Med.	Avg.	Med.	Avg.	Med.	Avg.	Med.	Avg.	Med.	Avg.	Med.
Reference	−6.36	−6.46	−6.71	−6.49	−7.45	−7.26	−	−	0.48	0.47	0.73	0.74	−	−
liGAN^∗^	−	−	−	−	−6.33	−6.20	21.1%	11.1%	0.39	0.39	0.59	0.57	0.66	0.67
GraphBP^∗^	−	−	−	−	−4.80	−4.70	14.2%	6.7%	0.43	0.45	0.49	0.48	**0.79**	**0.78**
AR-SBDD	−5.75	−5.64	−6.18	−5.88	−6.75	−6.62	37.9%	31.0%	0.51	0.50	0.63	0.63	0.70	0.70
Pocket2Mol	−5.14	−4.70	−6.42	−5.82	−7.15	−6.79	48.4%	51.0%	0.56	0.57	**0.74**	**0.75**	0.69	0.71
TargetDiff	−5.01	−5.69	−6.33	−6.47	−7.62	−7.64	56.3%	57.3%	0.48	0.48	0.59	0.58	0.71	0.71
PMDM	−5.65	−5.63	−6.35	−6.39	−7.60	−7.58	59.4%	62.7%	**0.59**	**0.60**	0.58	0.59	0.70	0.70
NucleusDiff (ours)	**−6.12**	**−6.80**	**−6.93**	**−6.85**	**−7.90**	**−7.76**	**60.1%**	**63.0%**	0.39	0.39	0.53	0.53	0.74	0.73

The symbols (↑) and (↓) indicate a higher or lower value of the metric is preferable, respectively. “Avg.” and “Med.” represent average and median values, respectively. Due to incompatibility between certain atom types produced by liGAN (18) and GraphBP (5) and the parsing capabilities of AutoDock Vina, we employ QVina (21) to conduct the docking simulations for these two methods. The bold values indicate the best (top-ranked) performance among all methods for each metric, while the underlined values indicate the second-best performance.

We note that NucleusDiff surpasses all baseline models on 8 out of 14 evaluated metrics, with the exceptions of QED, SA, and Diversity. In Table 2, NucleusDiff is only surpassed by GraphBP (5) in terms of Diversity, yet it exhibits superior performance compared to another diffusion model, TargetDiff (7). According to the average Vina Dock, NucleusDiff generates molecules with high affinities to the pockets (−7.90), which is 6.43% better than the best autoregressive model baseline, AR-SBDD (4), and 22.16% better than the best diffusion model baseline, TargetDiff (7). Besides, NucleusDiff surpasses AR-SBDD (4) and TargetDiff (7) on average High Affinity (60.1%) by 58.6% and 6.7%, and average Diversity (0.74) by 6.71% and 4.23%. On the other hand, the SA of generated molecules should fall within a reasonable range so that the ability to explore the molecular space confined by protein pockets is high enough to discover potential molecules. As shown in Table 2, the average QED of NucleusDiff (0.39) is slightly lower than that of AR-SBDD (4) (0.51) and TargetDiff (7) (0.48), but remains comparable to that of liGAN (18) (0.39), implying that NucleusDiff satisfies this desired property. Notably, the molecules generated by NucleusDiff perform even better than those in the test set on Vina Score, Vina Min, and Vina Dock, suggesting that NucleusDiff has great potential to generate more drug-like molecules with higher affinity. NucleusDiff models the constraints between atomic nuclei and their surrounding manifold to prevent atomic collision, which is important for the generation of high-affinity and realistically viable pharmaceuticals. Although TargetDiff (7), a diffusion-based model, also generates molecules by sampling from a learned distribution, it solely considers the positional information of atomic nuclei when learning the distribution of atoms, which does not incorporate geometrical constraints to maintain reasonable atomic distances. Consequently, it is reasonable to assert that the NucleusDiff model, as a geometric diffusion generative model incorporating the manifold constraints, provides critical insights for the generation of molecules and pharmaceuticals with high binding affinities.

### 2.7. Case Study of NucleusDiff on COVID-19 Target.

Scientists and drug designers are particularly interested in how deep learning models generalize to real-world problems, where practical constraints and biological variability should be considered. To this end, we follow previous work and conduct a case study focused on a COVID-19-related therapeutic target (11). This case study allows us to assess the practical utility of NucleusDiff in addressing critical and timely challenges in structure-based drug design. In this context, we generate molecules targeting the COVID-19 therapeutic target and subsequently evaluate their binding affinities and drug-likeness properties using the same set of metrics discussed earlier, including the Vina Score, Vina Min, Vina Dock, QED, SA, and Diversity. Last, we assess the performance of NucleusDiff and TargetDiff on this COVID-19-related therapeutic target using the proposed PLCR metric.

#### 2.7.1. Atomic collision evaluation.

To evaluate the performance of NucleusDiff on the COVID-19 therapeutic target, we conduct a comprehensive comparison with TargetDiff. Both methods utilize diffusion-based models, enabling a thorough understanding of atomic collisions during the inference process of DDPM (14). Our evaluation involves the 3CL protease as a real-world therapeutic target, with its experimentally validated active ligands. For both NucleusDiff and TargetDiff, we set an identical number of timesteps (1,000) for inference. We analyze the collision ratio metric PLCR at 11 timesteps, sampled every 100 steps from Step-0 to Step-1000, and the main results are summarized in Table 1. We observe that TargetDiff shows a significant reduction in atomic collisions from Step-0 to Step-1000. However, NucleusDiff maintains a consistently lower collision ratio throughout the inference steps. Notably, NucleusDiff outperforms TargetDiff in the PLCR metric, achieving improvements of up to 66.7%. Specifically, in the final sampling steps, NucleusDiff achieves an almost negligible atomic collision ratio, verifying the effectiveness of our design.

Fig. 2 visualizes the collision ratio for both methods across the COVID-19 target (3CL), revealing a marked contrast in the convergence trends. NucleusDiff demonstrates a more pronounced and rapid reduction in collision ratios compared to TargetDiff, underscoring its potential for addressing challenging targets in real-world drug design, such as those encountered in COVID-19 research.

#### 2.7.2. Binding affinity evaluation.

We compare the performance of NucleusDiff and TargetDiff on a curated benchmark involving the COVID-19 therapeutic target 3CL, assessing the 14 metrics. As shown in Table 3, NucleusDiff outperforms TargetDiff on 8 out of 14 metrics. Specifically, NucleusDiff achieves a superior average Vina Score of −5.85 compared to TargetDiff’s −4.82, indicating a stronger binding affinity. NucleusDiff also excels in the Vina Min and Vina Dock metrics, with average scores of −6.21 and −6.74, respectively, compared to −5.61 and −6.39 for TargetDiff. In the context of high-affinity ligands, NucleusDiff generated 70.0% high-affinity ligands versus 50.5% for TargetDiff, showing a clear advantage. Although NucleusDiff has a slightly lower average QED score (0.43) compared to TargetDiff (0.56), it still maintains acceptable drug-likeness properties. Additionally, both models exhibit similar performance in synthetic accessibility (SA) and molecular diversity, with NucleusDiff achieving average scores of 0.54 and 0.73, respectively, slightly below TargetDiff’s values of 0.62 and 0.76. These results demonstrate that NucleusDiff is more effective in generating high-affinity ligands while maintaining a balance with other drug design criteria, making it a strong candidate for real-world therapeutic applications, particularly those related to COVID-19. We further assess the generalization capabilities of NucleusDiff and TargetDiff on two additional therapeutic targets, with detailed results and analyses provided in *SI Appendix* due to space limitations.

**Table 3.** A summary of 14 biochemical properties for molecules generated by TargetDiff and NucleusDiff for target 3CL

	Vina score (↓)		Vina min (↓)		Vina dock (↓)		High affinity (↑)		QED (↑)		SA (↑)		Diversity (↑)	
Metrics	Avg.	Med.	Avg.	Med.	Avg.	Med.	Avg.	Med.	Avg.	Med.	Avg.	Med.	Avg.	Med.
TargetDiff	−4.82	−5.08	−5.61	−5.68	−6.39	−6.49	50.5%	50.5%	**0.56**	**0.54**	**0.62**	**0.61**	**0.76**	**0.76**
NucleusDiff (ours)	**−5.85**	**−5.80**	**−6.21**	**−6.23**	**−6.74**	**−6.84**	**70.0%**	**70.0%**	0.43	0.42	0.54	0.53	0.73	0.73

The symbols (↑) and (↓) indicate a higher or lower value of the metric is preferable, respectively. “Avg.” and “Med.” represent average and median values, respectively. The bold values indicate the best (top-ranked) performance among all methods for each metric.

### 2.8. The Results for Minimum Distance Constraint.

Our research is dedicated to addressing the critical challenge of atomic collisions in structure-based drug design. While NucleusDiff incorporates soft constraints during training to mitigate atomic collisions, an alternative approach involves implementing minimum distance constraints during the sampling process of pretrained models. In this part, we rigorously evaluate the efficacy of applying minimum distance constraints to the sampling process of pretrained NucleusDiff and TargetDiff models, assessing the generated molecules’ performance in terms of both atomic collision performance and binding affinity.

Given that NucleusDiff has demonstrated near-elimination of atomic collision on the CrossDocked2020 dataset, we conduct a more representative experiment. This involves examining the properties of 1,000 molecules sampled from both NucleusDiff and TargetDiff models using a minimum distance constraint inference process, specifically targeting the COVID-19 target (3CL). This approach enables a more thorough evaluation of the models’ capabilities under minimum distance constraint inference conditions, providing deeper insights into their performance in this context.

#### 2.8.1. Minimum distance constraint for the inference process of TargetDiff and NucleusDiff.

The high-level idea of the minimum distance constraint is to correct the distances of atom pairs that exhibit atomic collisions during the inference process. In this paper, we present two types of minimum distance constraints as postcorrection schemes:

##### 2.8.1.1. Minimum distance constraint (parallelogram).

For protein–ligand atom pairs (a,b) with atomic collision, we first identify atom *c* within the ligand that is closest to ligand atom *b*. Clearly, the line connecting the protein–ligand pair in 3D space intersects with a sphere centered at ligand atom *c*, with the covalent radius equal to the distance between *c* and *b*. One intersection point obviously becomes *b*, while the other intersection point $b^{\prime }$ becomes the corrected position of *b* after applying the minimum distance constraint.

##### 2.8.1.2. Minimum distance constraint (circle).

For a protein–ligand pair (a,b) exhibiting atomic collision, we first identify the atom *c* within the ligand that is closest to ligand atom *b*. We ensure that the distance between *a* and *b* is less than that between *a* and *c*. Based on this condition, we construct two spheres: one centered at protein atom *a* with a radius equal to the covalent radius of *a* and *b* (noting that the distance between (a,b) is less than the summation of their covalent radius) and another centered at ligand atom *c* with a radius equal to the distance between *c* and *b*. If these two spheres intersect, their intersection forms a circular region. By deriving the analytical expression for this circle, we sample a new point $b^{\prime }$ on this circle using a predetermined random seed (42). This $b^{\prime }$ effectively avoids atomic collisions while preserving the ligand’s geometric characteristics. In the case where the two spheres are tangent, the point of tangency serves as the unique corrected position $b^{\prime }$, avoiding atomic collisions and maintaining the ligand’s geometric properties.

#### 2.8.2. The atomic collision evaluation for minimum distance constraint.

The experimental results present in Table 4 demonstrate the efficacy of implementing minimum distance constraints to mitigate atomic collisions in structure-based drug design, specifically for the COVID-19 target (3CL). We evaluate the performance using the proposed PLCR metric. For TargetDiff, the baseline model without minimum distance constraints exhibits a nonnegligible level of atomic collisions, with 5 collisions per 210,000 atom pairs (PLCR). Notably, the implementation of both parallelogram and circle minimum distance constraints completely eliminates these collisions over the PLCR metric, resulting in zero collisions for all ratios. Similarly, NucleusDiff shows a slight improvement in the baseline performance compared to TargetDiff, with 3 collisions per 210,000 atom pairs (PLCR). This baseline improvement can be attributed to the manifold-constrained modeling approach inherent to NucleusDiff. Nevertheless, the application of minimum distance constraints (both parallelogram and circle methods) yields the same perfect results as observed with TargetDiff, completely eliminating all atomic collisions.

**Table 4.** The PLCR performance among pocket–ligand pairs for structure-based drug design of TargetDiff and NucleusDiff in the COVID-19 target

Models	TargetDiff	NucleusDiff (ours)
-	5/210000	3/210000
+ Parallelogram	0/210000	0/210000
+ Circle	0/210000	0/210000

Two types of minimum distance constraints are considered: w/ Parallelogram and w/ Circle.

These results underscore the validity of incorporating minimum distance constraints in the sampling process of pretrained models for structure-based drug design. Both the parallelogram and circle constraint methods prove equally effective in resolving atomic collisions, suggesting that either approach can be reliably employed to enhance the physical realism of generated molecular structures. The complete elimination of atomic collisions over the PLCR metric for both TargetDiff and NucleusDiff when using minimum distance constraints highlights the robustness of this approach. This improvement is particularly significant for the COVID-19 target (3CL), demonstrating the potential of these methods in generating more physically viable drug candidates for this crucial therapeutic target.

#### 2.8.3. The binding affinity evaluation for minimum distance constraint.

In this study, we analyze the effects of incorporating a minimum distance constraint to the model’s inference process, which influences the physicochemical properties of atomic-collision molecule generated by TargetDiff and NucleusDiff for the COVID-19 target, 3CL. We select one representative atomic-collision molecule each for TargetDiff and NucleusDiff, with the experimental results detailed in Table 5.

**Table 5.** The binding affinity performance of atomic-collision molecule generated by TargetDiff and NucleusDiff for target 3CL

Method	Vina score (↓)	Vina min (↓)	Vina dock (↓)
TargetDiff	19.287	−0.543	−6.393
+ Parallelogram	20.124	−0.363	−6.387
+ Circle	20.270	−1.827	−6.075
NucleusDiff (ours)	−5.946	−7.055	−7.646
+ Parallelogram	Invalid	Invalid	Invalid
+ Circle	−5.939	−6.516	−6.441

Two types of minimum distance constraints are considered: w/ Parallelogram and w/ Circle. The symbols (↑) and (↓) indicate whether a higher or lower value of the metric is preferable, respectively.

The results prominently demonstrate that enforcing minimum distance constraints often leads to a decrease in binding affinity, a critical factor in drug design. For the atomic-collision molecule generated by Targetdiff, this trend is clearly observed. The Vina Score, a key indicator of binding affinity where lower values are more favorable, increases from 19.287 in the unconstrained version to 20.124 with the parallelogram constraint and 20.270 with the circle constraint. This consistent increase in Vina Score across both constraint methods signifies a reduction in binding affinity. The atomic-collision molecule generated by NucleusDiff further corroborates this trend, albeit to a lesser extent. While the parallelogram constraint results in an invalid structure, the circle constraint method produces a valid molecule with a slightly higher Vina Score (−5.939) compared to the unconstrained version (−5.946). Although this difference is minimal, it still aligns with the overall trend of decreased binding affinity when constraints are applied.

These findings underscore a crucial trade-off in the application of minimum distance constraints: While they effectively address atomic collisions, they often do so at the cost of reduced binding affinity. This phenomenon was consistently observed across both TargetDiff and NucleusDiff methods, indicating that it may be a general consequence of applying such constraints rather than a method-specific effect. The observed decrease in binding affinity highlights the need for careful consideration when applying minimum distance constraints in structure-based drug design. While these constraints serve an important purpose in eliminating atomic collisions, their potential to compromise binding affinity could have significant implications for drug efficacy.

### 2.9. Visual Analysis of NucleusDiff on CrossDocked2020 and COVID-19 Target.

**Fig. 3.**
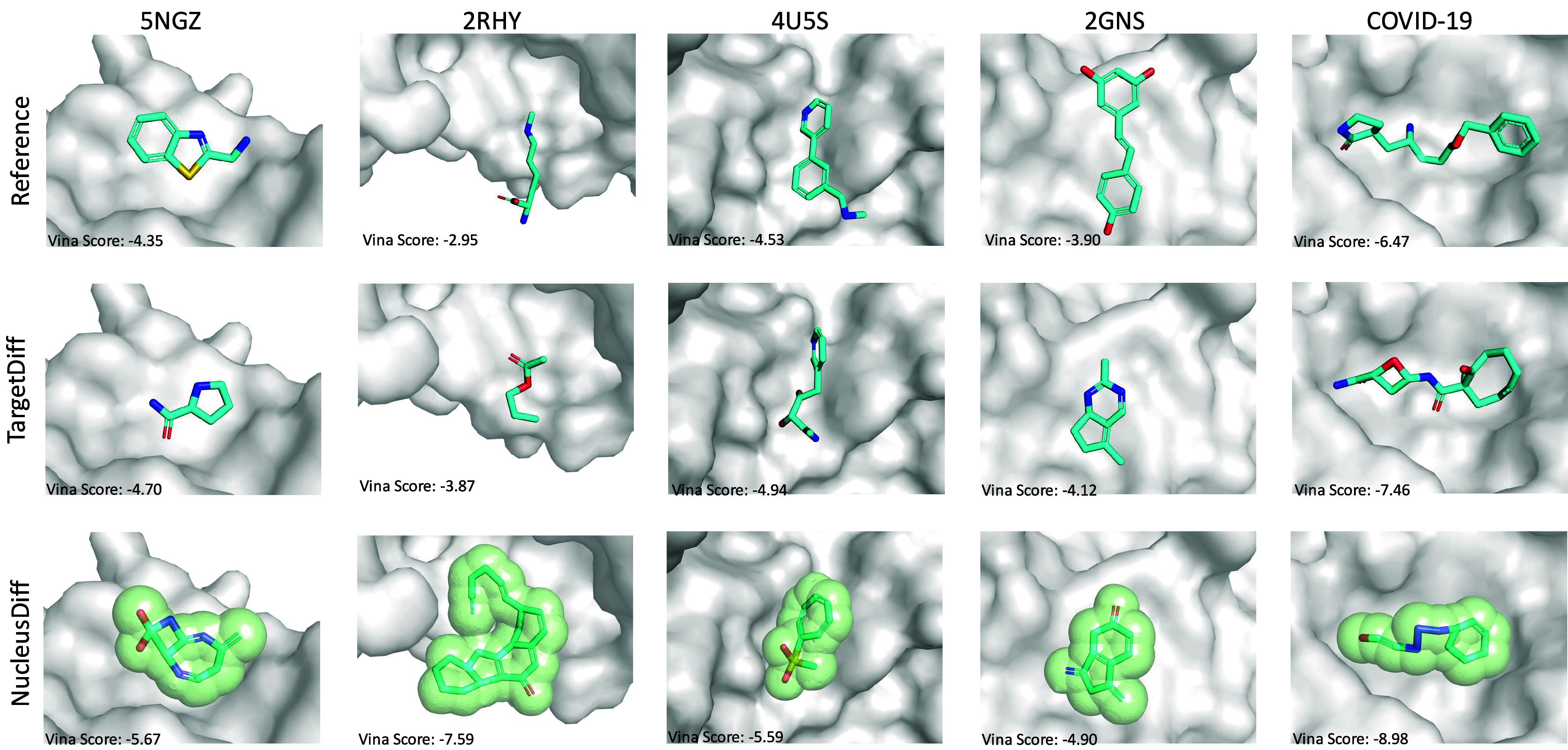
Visualization of the pockets and sampled ligands on CrossDocked2020 and COVID-19. The sampled molecules are generated using TargetDiff and NucleusDiff. For NucleusDiff, we illustrate both the generated nucleus and manifold (marked in the green sphere). We also emphasize the use of the Vina Score to measure the quality of generated ligands, where a lower score indicates stronger binding affinity.

Fig. 3 illustrates the ligands generated by NucleusDiff and TargetDiff, alongside reference ligands, for specific binding pockets. We select five representative protein pockets for structural analysis: 5NGZ, 2RHY, 4U5S, 2GNS from the CrossDocked2020 dataset, and an additional COVID-19 target. Both TargetDiff and NucleusDiff demonstrate the capability to generate structurally diverse ligands that conform to their respective binding pockets. Notably, NucleusDiff consistently produces ligands with superior Vina Scores compared to both TargetDiff and the reference ligands across all examined pockets. This is particularly evident for the COVID-19 target, where NucleusDiff achieves a remarkable Vina Score of −8.98, substantially outperforming TargetDiff (−7.46) and the reference ligand (−6.47). A key distinction between TargetDiff and NucleusDiff lies in the molecular representations they generate. While TargetDiff generates only atomic positions, NucleusDiff provides a more comprehensive visualization by not only displaying atomic nuclei positions but also incorporating the manifold of generated molecules. This is depicted by a green spherical manifold encompassing the ligand structure, providing a deeper understanding of how the molecule occupies space within the binding pocket.

It is important to note that our evaluation of molecular properties focuses on metrics such as binding affinity (Vina score, Vina Min, and Vina Dock), QED (Quantitative Estimate of Drug-likeness), SA (Synthetic Accessibility), and Diversity. Structural similarity between the generated molecule and the reference ligand is not necessarily required, as the generative model is not designed to replicate these structures exactly. Also, the reference ligand is not always the optimal drug candidate for the target, and its structure often leaves room for improvement. As these reference structures are typically derived through computational chemistry methods, the ability of the generative model to produce diverse ligands is a significant advantage.

Due to space limitations, the technical details of the manifold generation process are elaborated in *SI Appendix*. However, the manifold representation in NucleusDiff visualizations offers crucial insights: 1) It enables the reconstruction of the ligand’s manifold, demonstrating that the model has learned to generate structures that adhere to van der Waals radius constraints-a key physical property that is incorporated into our objective function. 2) This approach sets our work apart from previous structure-based drug design efforts by explicitly modeling and visualizing the manifold distribution, rather than focusing solely on atomic nuclei positions. The clear delineation of ligand boundaries within protein pockets, especially evident in the model’s representations, suggests that NucleusDiff not only effectively learns the relative positioning of ligands within pockets but also maintains proper geometric constraints to prevent atomic overlaps. This comprehensive approach likely contributes to the generation of ligands with improved binding affinities and a reduced likelihood of atomic collision constraints.

## 3. Conclusion

In this work, we first introduce three metrics to effectively quantify the atomic collision issue of existing deep generative models for ligand design in structure-based drug discovery tasks. We then present NucleusDiff, which jointly models the atomic nucleus and the surrounding manifold to address this collision issue. Empirical results reveal that NucleusDiff not only achieves superior performance on existing stability and potency metrics but also significantly mitigates atomic collisions and converges more rapidly to the target geometric distribution.

One limitation of NucleusDiff is its current focus on modeling the ligand manifold while neglecting the protein manifold. Incorporating the protein manifold into the ligand manifold is an important aspect that we aim to explore in future work. Additionally, NucleusDiff utilizes a discretized version of the manifold, represented by triangle mesh points. Yet in reality, the manifold is continuous. This requires a more profound understanding and utilization of the first-principle theorem, and we would like to leave this for future research.

## 4. Materials and Methods

Our main goal is to jointly model the nucleus and the manifold over the electron cloud surrounding each nucleus, aiming to reduce the atomic collision issue in the diffusion model sampling process for structure-based drug design. We first explain how the DDPM is applied to the existing structure-based drug design modeling. Following this, we introduce how we adopt the manifold-constrained modeling in NucleusDiff. Last but not least, we provide more details on the training objective and inference, along with insights into the architecture specifics.

### 4.1. Nucleus Diffusion for Atomic Nuclei Generation.

Here, our main goal is to model the nuclei types $v^{N}$ and nuclei coordinates $x^{N}$ given the protein pocket $\left(v^{P},x^{P}\right)$: $p(v^{N},x^{N}|v^{P},x^{P})$. We follow the existing DDPM for the structure-based drug design pipeline by estimating this conditional with a categorical diffusion model on atomic types and a continuous diffusion model on atomic coordinates (7, 22).

In the main manuscript, we define the variance scheduler $\beta_{t}$ and $\alpha_{t}$, and how to derive the prior $q(x_{t}^{N}|x_{0}^{N})$ and posterior $q(x_{t-1}^{N}|x_{t}^{N},x_{0}^{N})$ for nuclei coordinates at time *t*. Similarly, for the nuclei types, we use categorical distribution *C*, and suppose we have *K* nuclei types in total. The prior distribution and posterior distribution of nuclei types at time *t* are

[9] $$\begin{array}{c} \begin{array}{cc} q(v_{t}^{N}|v_{0}^{N}) & =C(v_{t}^{N}|{\overset{¯}{\alpha }}_{t}v_{0}^{N}+\left(1-{\overset{¯}{\alpha }}_{t}\right)/K),\\ q(v_{t}^{N}|v_{t}^{N},v_{0}^{N}) & =C(v_{t}^{N}|\theta_{c}\left(v_{t}^{N},v_{0}^{N}\right)), \end{array} \end{array}$$

where $\theta_{c}\left(v_{t}^{N},v_{0}^{N}\right)=\theta^{*}/\sum_{k}\theta_{k}^{*}$, and $\theta^{*}=\left[\alpha_{t}v_{t}^{N}+\left(1-\alpha_{t}\right)/K\right]⊙\left[{\overset{¯}{\alpha }}_{t-1}v_{0}^{N}+\left(1-{\overset{¯}{\alpha }}_{t-1}\right)/K\right]$, where ⊙ is element-wise product. Then after reparameterization, we predict ${\hat{v}}_{0}^{N}$ from $v_{t}^{N}$, *i.e.*, ${\tilde{v}}_{0}^{N}=\mu_{\theta }\left(v_{t}^{N},t\right)$. Injecting this into the posterior, then the objective functions for the discrete types and continuous coordinates are

[10] $$\begin{array}{c} \begin{array}{cc} L_{t-1}\left(v^{N}\right) & =\;\text{KL}(q\left(v_{t}^{N}|v_{t}^{N},v_{0}^{N}\right)||q\left(v^{N}|{\tilde{v}}_{0}^{N}\right))\\ & =\sum_{k}\theta_{c}{\left(v_{t}^{N},v_{0}^{N}\right)}_{k}\cdot \frac{\theta_{c}{\left(v_{t}^{N},v_{0}^{N}\right)}_{k}}{\theta_{c}{\left(v_{t}^{N},{\tilde{v}}_{0}^{N}\right)}_{k}},\\ L_{t-1}\left(x^{N}\right) & =E_{q}[{\left\|x_{0}^{N}-{\hat{x}}_{0}^{N}\left(x_{t}^{N},t,v^{P},x^{P}\right)\right\|}^{2}]. \end{array} \end{array}$$

### 4.2. Manifold-Constraint Denoising Diffusion Model.

Meanwhile, the generated nuclei coordinates should adhere to reasonable geometric properties: Atoms are not treated as solid points but consist of nuclei surrounded by spatial constraints; thus, there exists a minimum distance between pairwise atoms. Ignoring this can lead to the atomic collision issue. To address this, we jointly model the geometric constraints of the manifold and the atomic nuclei for structure-based drug design. To be more concrete, for each nucleus, we construct a discrete manifold, where the radius is the van der Waals radius *R*. Then for each nucleus, we obtain its *c* closest mesh points in the manifold, marked as $x^{M}$. Thus, instead of $p(v^{N},x^{N}|v^{P},x^{P})$, the objective is to maximize the following likelihood $p(v^{N},x^{N},x^{M}|v^{P},x^{P})$. The objective on the manifold at time *t* is Eq. **5**.

On the other hand, recall that the mesh points are scattered around the nuclei with van der Waals radius *R*. Motivated by this, we add a regularization term by forcing the distance between each mesh point and nuclei to be close to *R* as in Eq. **6**.

### 4.3. Learning and Inference.

To sum up, the training objective function is composed of four parts, as in Eq. **8**. For the inference, because the mesh points from manifold modeling are treated as the auxiliary components of the physics-guided nuclei modeling, they can be ignored at this stage, while only the nuclei coordinates are considered. We provide the detailed pseudocodes for inference in *SI Appendix*.

### 4.4. Computational Resources.

All algorithms and models have been developed using Python 3.8.13, with PyTorch version 1.12.1 and PyTorch Geometric version 2.5.2, under CUDA 11.0. Experiments are conducted on a server with 8 NVIDIA V100 GPUs (32 GB memory) and Intel(R) Xeon (R) Platinum 8255C CPU @ 2.50GHz. We employ a single V100 GPU for training while leveraging eight GPUs to accelerate the sampling procedure. The models typically converge after approximately 48 h of training and sampling 10 K ligands using eight GPUs takes about 12 h.

## Supplementary Material

Appendix 01 (PDF)

Movie S1. A demo illustrating the inference process of the TargetDiff and NucleusDiff.

## Data Availability

Code and raw data have been deposited in GitHub (https://github.com/yanliang3612/NucleusDiff) (23). We provide the code and dataset generation scripts at this GitHub repository. The code developed for this study is released under the MIT License.

## Footnotes

^*^These parameters are specific to dataset preprocessing rather than the core model architecture, and thus we consider a full sensitivity analysis to be unnecessary.
