# Supplementary material for: Manifold-constrained nucleus-level denoising diffusion model for structure-based drug design
Source: Proc Natl Acad Sci U S A. 2025 Oct 6;122(41):e2415666122. doi: 10.1073/pnas.2415666122 (PMC12541315; doi:10.1073/pnas.2415666122)
Supplement: Supplementary file 1 — Appendix 01 (PDF) [file pnas.2415666122.sapp.pdf]

Supporting Information for

Manifold-Constrained Nucleus-Level Denoising Diffusion Model for Structure-Based Drug Design

Shengchao Liu, Liang Yan, Weitao Du, Weiyang Liu, Zhuoxinran Li, Hongyu Guo, Christian Borgs, Jennifer Chayes, Anima Anandkumar

E-mail: anima@caltech.edu

This PDF file includes:

Supporting text
Figs. S1 to S11
Tables S1 to S21
Legend for Movie S1
SI References

Other supporting materials for this manuscript include the following:

Movie S1

Supporting Information Text

1. Related Work

Structure-based Drug Design. In recent years, the availability of structural data catalyzes the development of numerous generative models for the *target-aware* molecule generation task. Such models include those by (1, 2), which generate SMILES representations based on protein contexts, and the flow-based model proposed by (3) for generating molecular graphs conditional on protein target sequence embeddings. (4) explores the generation of 3D molecules through the voxelization of molecules in atomic density grids within a conditional VAE framework. (5) leverages normalizing flow to generate pocket-based 3D molecules, with properties controlled by a tailored prior distribution. Further, (6) employs Monte-Carlo Tree Search coupled with a policy network for optimizing molecules in 3D space. Notably, (7–9) develop autoregressive models for atom-by-atom 3D molecule generation using Graph Neural Networks (GNNs). Despite these advancements, current models still grapple with several challenges. These include the separate encoding of small molecules and protein pockets (1–4), reliance on voxelization techniques and non-equivariant networks (1, 2, 4), and the limitations inherent to autoregressive sampling methods (7–9). In contrast to previous approaches, our equivariant diffusion-based generative model innovatively integrates 3D protein-ligand interactions within a unified framework while employing non-autoregressive sampling, thereby enhancing the model's capacity to capture complex molecular relationships and improving the consistency between training and inference processes.

Molecular and Protein Manifold Learning. Manifold learning has been widely applied in the vision tasks (10–14). Additionally, recent advancements in manifold learning for molecular & protein have garnered attention in the scientific community. Several studies (15–18) embark on an innovative trajectory by employing or integrating sophisticated representational learning techniques pertaining to molecular and protein surfaces. This approach facilitates a precise articulation and comprehension of the intricate complexities inherent in molecular structures.(15) introduces MaSIF, a method using deep learning to identify and predict how proteins interact with other molecules by analyzing patterns on their surfaces. SurfGen (16), introducing its two neural networks, Geodesic-GNN and Geoatom-GNN, effectively analyzes topological interactions on pocket surfaces and spatial interactions between ligand atoms and surface nodes for advanced molecular prediction. HMR (17) employs Laplace-Beltrami eigenfunctions for representing molecules on 2D Riemannian manifolds, enhancing molecular encoding through harmonic message passing. Atomsurf (18) explores the use of 3D mesh surfaces for representing proteins, revealing that while promising, this method alone is less effective than 3D grids. It proposes a novel framework that synergistically combines surface representations with graph-based methods for improved protein representation learning.

2. Formulation and Analysis of Proposed Atomic Collision Metrics

The phenomenon of atomic collision arises when two atoms come sufficiently close to each other such that their electron clouds overlap. This overlap violates the Pauli exclusion principle and the electrostatic repulsion between electrons. To quantify this proximity, we employ covalent radius, denoted as d. Consider a scenario involving a ligand atom with coordinates $\boldsymbol{x}_i$ and a protein atom with coordinates $\boldsymbol{x}_j$. The respective covalent radii for these atoms are d_i and d_j. During the ligand generation sampling process, if the distance between these two atoms becomes excessively small, specifically if $\|\boldsymbol{x}_i - \boldsymbol{x}_j\| \leq D_{ij} = d_i + d_j$, we classify this event as an *atomic collision*. To systematically analyze and understand this phenomenon, we propose three distinct metrics for its quantification, each derived from different levels of assessment granularity. These metrics provide a comprehensive framework for evaluating the occurrence and implications of atomic collisions in molecular simulations and structural biology studies.

Pairwise-Level Collision Ratio (PLCR). The first metric we introduce is the atom **Pairwise-Level Collision Ratio (PLCR)**. This metric quantifies the collision ratio between ligand atoms and their nearby protein atoms within the binding site. The PLCR provides a detailed measure of how often atomic collisions occur at the pairwise level between ligand and protein atoms within a binding site. For each ligand atom, denoted as $\boldsymbol{x}_i^L$, we identify its K nearest protein atoms within the binding site. This step ensures that we consider only the most relevant interactions in the context of the ligand-protein binding interface. The PLCR is then defined mathematically as follows:

$$\text{PLCR} = \frac{\sum_{k \in N_{\text{mol}}, i \in N^k_{\text{atom}}, j \in N^i_{\text{nearest}}} \mathbb{1}(\|\boldsymbol{x}_i - \boldsymbol{x}_j\| < D_{ij})}{K \cdot \sum_{k \in N_{\text{mol}}} N^k_{\text{atom}}}, \quad [1]$$

where the variables are defined as:

- N_{mol}: The total number of ligand molecules.
- N^k_{atom}: The number of atoms in the k-th ligand molecule.
- N^i_{nearest}: The number of the nearest protein atoms to the i-th ligand atom.
- $\mathbb{1}(\cdot)$: The indicator function, which returns 1 if the condition inside is true (i.e., if the distance between the i-th ligand atom and the j-th protein atom is less than D_{ij}), and 0 otherwise.
- $\|\boldsymbol{x}_i - \boldsymbol{x}_j\|$: The Euclidean distance between the coordinates of the i-th ligand atom and the j-th protein atom.
- D_{ij}: The sum of the covalent radius of the i-th ligand atom and the j-th protein atom.

The PLVR metric provides a normalized measure of the frequency of atomic collisions, which allows for a comparative analysis across different ligand-protein systems. This metric is particularly useful for assessing the spatial compatibility of ligands within protein binding sites, and it can be used to guide the optimization of ligand design to minimize unfavorable atomic interactions. The PLVR metric provides a rigorous and systematic evaluation of atomic collisions. This facilitates more accurate and informative assessments in molecular simulations and structural biology research.

Atom-Level Collision Ratio (ALCR). The second metric we propose is the **Atom-Level Collision Ratio (ALCR)**. This metric provides a more aggregated view by focusing on individual ligand atoms and determining whether they are involved in any collisions with protein atoms. The ALCR measures the proportion of ligand atoms that have at least one of their K nearest protein atoms within a critical distance. This critical distance is defined as less than the sum of their respective covalent radii.

More rigorously, an atom is flagged as involved in a collision if any one of its K nearest neighboring protein atoms is within this critical distance threshold. The ALCR provides a broader perspective on the collision propensity of each ligand atom, moving beyond pairwise interactions to a more holistic assessment.

The ALCR is mathematically defined as follows:

$$\text{ALCR} = \frac{\sum_{k \in N_{\text{mol}}, i \in N^k_{\text{atom}}} \mathbb{1}\left(\sum_{j \in N^i_{\text{nearest}}} \mathbb{1}(\|\boldsymbol{x}_i - \boldsymbol{x}_j\| < D_{ij}) > 0\right)}{\sum_{k \in N_{\text{mol}}} N^k_{\text{atom}}}, \quad [2]$$

where the variables are defined as:

- N_{mol}: The total number of ligand molecules.
- N^k_{atom}: The number of atoms in the k-th ligand molecule.
- N^i_{nearest}: The number of the nearest protein atoms to the i-th ligand atom.
- $\mathbb{1}(\cdot)$: The indicator function, which returns 1 if the condition inside is true and 0 otherwise.
- $\|\boldsymbol{x}_i - \boldsymbol{x}_j\|$: The Euclidean distance between the coordinates of the i-th ligand atom and the j-th protein atom.
- D_{ij}: The sum of the covalent radius of the i-th ligand atom and the j-th protein atom.

In this definition, the inner $\mathbb{1}(\cdot)$ function checks if the distance between the i-th ligand atom and any of its K nearest protein atoms is smaller than D_{ij}. If at least one such pair is found, the outer $\mathbb{1}(\cdot)$ function marks the i-th ligand atom as involved in a collision.

The ALCR provides a normalized measure of the frequency of atomic collisions at the atom level. This enables researchers to identify specific ligand atoms that are more prone to violations. The metric is particularly valuable for guiding ligand optimization processes, as it ensures that individual atomic interactions are considered in the context of the entire ligand-protein binding interface. The ALCR enables researchers to achieve a more nuanced understanding of collision dynamics, ultimately facilitating the design of more compatible and effective ligands in molecular simulations and structural biology studies.

Molecule-Level Collision Ratio (MLCR). The final metric we introduce is the **Molecule-Level Collision Ratio (MLCR)**. This metric applies the collision analysis to the entire molecule, providing a macroscopic view of collision occurrences. The MLCR quantifies the proportion of ligand molecules that exhibit at least one atomic collision within their structure. In essence, a molecule is marked as involved in a collision if any of its constituent atoms is flagged for an atom-level collision.

To compute the MLCR, we aggregate the atom-level collisions for each molecule. If any atom within a molecule meets the collision criteria (i.e., it has at least one of its K nearest protein atoms within the critical distance threshold), the entire molecule is considered to be in collision.

The MLCR is mathematically defined as follows:

$$\text{MLCR} = \frac{\sum_{k \in N_{\text{mol}}} \mathbb{1}\left(\sum_{i \in N^k_{\text{atom}}, j \in N^i_{\text{nearest}}} \mathbb{1}(\|\boldsymbol{x}_i - \boldsymbol{x}_j\| < D_{ij}) > 0\right)}{N_{\text{mol}}}, \quad [3]$$

where the variables are defined as:

- N_{mol}: The total number of ligand molecules.
- N^k_{atom}: The number of atoms in the k-th ligand molecule.
- N^i_{nearest}: The number of the nearest protein atoms to the i-th ligand atom.
- $\mathbb{1}(\cdot)$: The indicator function, which returns 1 if the condition inside is true and 0 otherwise.
- $\|\boldsymbol{x}_i - \boldsymbol{x}_j\|$: The Euclidean distance between the coordinates of the i-th ligand atom and the j-th protein atom.
- D_{ij}: The sum of the covalent radius of the i-th ligand atom and the j-th protein atom.

In this context, the inner $\mathbb{1}(\cdot)$ function checks if the distance between any pair of the i-th ligand atom and its K nearest protein atoms is smaller than D_{ij}. If at least one such pair is found, the outer $\mathbb{1}(\cdot)$ function flags the molecule as having a collision.

The MLCR provides a normalized measure of the frequency of atomic collisions at the molecule level. This metric captures the overall collision propensity of entire ligand molecules and is particularly valuable for identifying and optimizing ligand molecules with minimal collision tendencies. Consequently, it ensures better compatibility and stability when ligands bind to protein targets. The MLCR enables the researchers to achieve a comprehensive understanding of collision dynamics across different ligand molecules, thereby facilitating the design and selection of more effective and structurally compatible ligands in molecular design and structural biology studies.

Summary. The three defined metrics, PLCR (Pairwise-Level Collision Ratio), ALCR (Atom-Level Collision Ratio), and MLCR (Molecule-Level Collision Ratio), serve as pivotal indicators for assessing atomic collision between protein pockets and generated ligands. These metrics elucidate the machine learning inference process within the context of Structure-Based Drug Design (SBDD) tasks. Each metric provides a unique perspective: PLCR evaluates the ratio of collisional pairs to total atom pairs, ALCR examines the ratio of collisional atoms to total atoms, and MLCR assesses the ratio of collisional molecules to total molecules.

3. Details for Manifold Reconstruction

In this part, we provide a detailed description of the technical process employed for the manifold reconstruction from mesh points, as applied in this work. The reconstruction process includes several critical steps, as follows:

Identifying Proximal Mesh Points for Each Atom. The primary objective is to demonstrate the manifold constructed from the mesh points generated and sampled by our pre-trained model. To achieve this, we identify all mesh points that are in close proximity to each atom. By calculating the distance, we can obtain mesh points whose distances to the sampled atom are close to the van der Waals radius.

Manifold Reconstruction for Each Individual Atom. Using the `MeshLab` software, we calculate the mesh for each individual atom. Specifically, we employ the `Poisson reconstruction` function in `MeshLab` , which is crucial for generating a smooth and continuous surface from the scattered mesh points. This step is essential for visualizing the atomic structure and understanding the distribution of mesh points around each atom.

Addressing the Sampling Problem. A significant challenge is that the number of reasonable mesh points obtained from a single sampling is typically too small, resulting in unreasonable meshes for each atom. This issue requires a solution for effective manifold reconstruction. We address this problem using two main strategies. First, we employ multiple sampling to obtain a sufficient number of reasonable mesh points. These points are then combined into a single manifold. Different random seeds ensure variability and robustness in the sampling process. Second, we utilize data augmentation techniques to artificially increase the diversity and quantity of the data. These techniques include transformations such as scaling, rotation, and translation of the mesh points. Both strategies work in tandem to enhance the robustness of the manifold reconstruction, providing a more comprehensive and accurate representation of the atomic structure.

Fusing Different Meshes Using `TriMesh` Software. We utilize the `TriMesh` software to fuse the different meshes obtained from multiple sampling. The process involves loading the mesh files, filling any holes, updating faces to remove duplicates and degenerate faces, and validating the resultant mesh. `TriMesh` is particularly effective for handling complex mesh operations and ensuring that the final mesh is a closed volume.

Smoothing the Mesh and Final Reconstruction. Some areas of the mesh are too abrupt and require smoothing. We apply Laplacian smoothing in `MeshLab` to clean up the manifold, ensuring a more natural and accurate representation. This smoothing process helps eliminate artifacts and irregularities, resulting in a cleaner and more visually appealing mesh. The final reconstructed manifold effectively demonstrates the success of our manifold reconstruction process, illustrating both the atomic nuclei and the electron cloud. This comprehensive approach ensures a detailed and accurate representation of the atomic structure. It provides valuable insights into the spatial distribution and interactions within the molecule.

4. NucleusDiff's Details

A. Difference Between Manifold Constraint and Atomic Collision Constraint In Learning. We have explained how the three collision metrics are constructed in the main article and Section 2. A simple approach is to use these three metrics as regularization terms during modeling. However, we emphasize two main differences between using manifold constraints and atomic collision metrics as constraints, which is also visualized in Figure S1.

- **Physical Meaning.** The atomic collision metrics discussed so far are atom-wise, thus they can be viewed as from the classic physics aspect. However, the intuition behind the manifold constraint is the manifold learning on the electron clouds, which is quantum physics. We highlight that from this aspect, NucleusDiff using manifold-constraint opens a novel research paradigm by incorporating quantum physics for structure-based drug design.
- **Efficiency.** Suppose we have N atoms, then with the atomic collision constraint, we must ensure the minimum distance for each atom pair, where the complexity is $O(N^2)$. However, for the manifold constraint, we only need to guarantee the minimum distance between each atom and its electron cloud manifold, *i.e.*, the complexity if $O(NM)$ where M is the number of sampled mesh points on the manifold. In our experiments, we take $M = 3$, thus using manifold constraint in NucleusDiff is more computationally efficient than using atomic collision constraint in learning.

We also note that we can add the atomic collision constraint during inference, yet this leads to worse binding affinity performance. Please check Section 7 for more details and results.

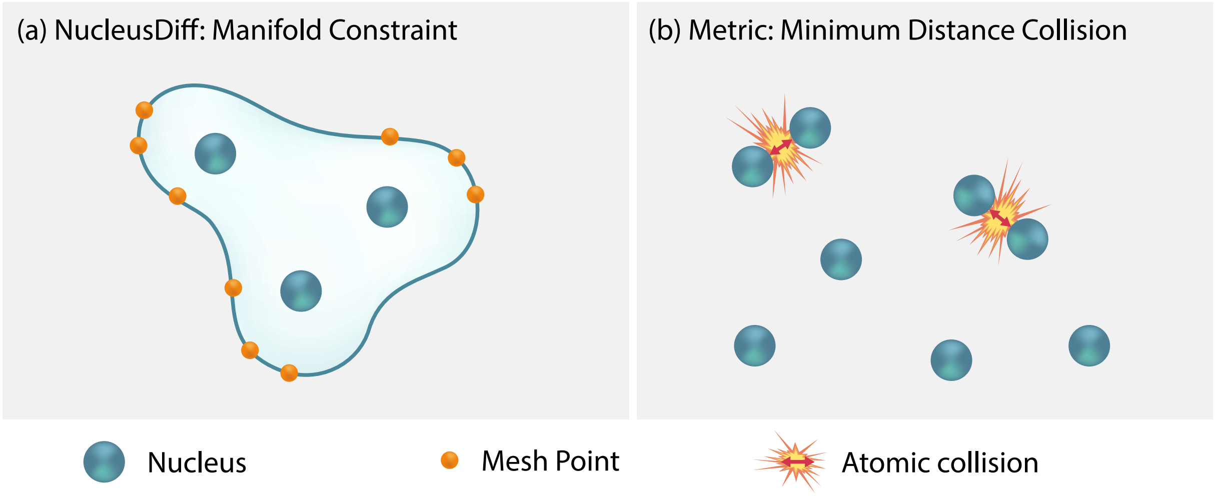

Fig. S1. The comparison of using manifold constraint and minimum distance constraint.

B. Architecture Details. In this subsection, we introduce details on the model architecture. The modeling on 3D atom positions needs to satisfy two important physical properties: such modeling should be equivariant to rotation and translation of the whole molecule system, *i.e.*, SE(3)-equivariance. Inspired by the recent advancements in equivariant neural networks (19–23), we use a SE(3)-Equivariant Graph Neural Network (24) to model the interactions between atoms of the ligand and the protein pocket, as well as the manifold of ligand:

$$[\hat{x}_0, \hat{h}_0] = \phi_\theta(\mathcal{M}_t, t, \mathcal{P}) = \phi_\theta([x_t, h_t], t, \mathcal{P}), \tag{4}$$

and

$$[\hat{v}_0, \hat{h}_0] = \phi_\theta(\mathcal{M}_t, t, \mathcal{P}) = \phi_\theta([v_t, h_t], t, \mathcal{P}). \tag{5}$$

In the l-th layer, we alternately update the atom's hidden embedding, denoted as h, and its coordinates, represented by x, in the following manner:

$$\begin{aligned} h_i^{l+1} &= h_i^l + \sum_{j \in \mathcal{V}, i \neq j} f_h(d_{ij}^l, h_i^l, h_j^l, e_{ij}; \theta_h) \\ x_i^{l+1} &= x_i^l + \sum_{j \in \mathcal{V}, i \neq j} (x_i^l - x_j^l) f_x(d_{ij}^l, h_i^{l+1}, h_j^{l+1}, e_{ij}; \theta_x) \cdot \mathbb{1}_{\text{mol}} \end{aligned} \tag{6}$$

We consider the distance d_{ij} as the Euclidean distance, which measures the straight line distance between two atoms, atom i and atom j. Additionally, $\mathbf{e}_{ij}$ is a feature that identifies whether the connection is between two protein atoms, two ligand atoms, or one of each. We also use a ligand molecule mask, denoted as $\mathbb{1}_{\text{mol}}$, to ensure that we only adjust the coordinates of ligand atoms, not protein atoms.

Initially, each atom is represented by a hidden embedding, $\mathbf{h}^0$, created by an embedding layer that incorporates atom-specific information. After processing through several layers, we obtain the final atom hidden embedding, $\mathbf{h}^L$. This embedding is then processed through a multi-layer perceptron and a softmax function to predict $\hat{\mathbf{v}}_0$.

An important aspect of this process ensures rotational equivariance. This means that if we rotate the initial atomic coordinates $\mathbf{x}_t$, the predicted coordinates $\hat{\mathbf{x}}_0$ will rotate in the same way. This is also true for the relationship between $\mathbf{x}_{t-1}$ and $\mathbf{x}_0$.

In summary, this process involves calculating distances between atoms, identifying atom types, and transforming atom information through several layers to predict new coordinates, all while maintaining consistency under rotation.

C. Algorithm. In this part, we present comprehensive details of the training and sampling process employed by NucleusDiff, which are delineated as Algorithm 1 for training and Algorithm 2 for sampling.

Algorithm 1 Training algorithm of NucleusDiff

Require: Protein-ligand binding dataset $\{\mathcal{P}, \mathcal{M}\}_{i=1}^{N}$, Ligand Manifold$\{\mathcal{V}_{i=1}^{K}, \mathcal{F}_{i=1}^{L}\}$, neural network ϕ_θ for modeling ligand distribution (parameterized by θ), ψ_θ for modeling mesh points distribution.

while ϕ_θ and ψ_θ not converge **do**

  Identifies the n closest mesh points for each atom $\{\mathcal{V}_{j=1}^{n}\}_{i=1}^{N}$, thereby obtaining the final dataset $\{\mathcal{P}, \mathcal{M}, \mathcal{V}_{j=1}^{n}\}_{i=1}^{N}$.

  Moves the complex (including the mesh points) to make center of mass (CoM) of protein atoms zero.

  Perturbs ligand postion $\boldsymbol{x}_0$ to obtain $\boldsymbol{x}_t$: $\boldsymbol{x}_{t_\gamma} = \sqrt{\bar{\alpha}_{t_\gamma}}\boldsymbol{x}_0 + (1-\bar{\alpha}_{t_\gamma})\epsilon$, where $\epsilon \in N(0, \boldsymbol{I})$.

  for the mesh points' postions $\boldsymbol{v}^i$ in $\{\mathcal{V}_{i=1}^{n}\}$ **do**

    Perturbs $\boldsymbol{v}_0^i$ to obtain $\boldsymbol{v}_{t_\tau}^i$: $\boldsymbol{v}_{t_\tau}^i = \sqrt{\bar{\alpha}_{t_\tau}}\boldsymbol{v}_0^i + (1-\bar{\alpha}_{t_\tau})\epsilon$, where $\epsilon \in N(0, \boldsymbol{I})$,

  Perturbs atom feature $\boldsymbol{f}_0$ to obtain $\boldsymbol{f}_{t_\gamma}$:

  $\log c = \log\left(\bar{\alpha}_{t_\gamma}\boldsymbol{f}_0 + (1-\bar{\alpha}_{t_\gamma})/K\right)$,

  $\boldsymbol{f}_{t_\gamma} = \texttt{one\_hot}(\mathrm{argmax}_i[g_i + \log c_i])$, where $g \sim \mathrm{Gumbel}(0,1)$,

  Uniformly encode the mesh points as $\boldsymbol{h}_0$ to distinguish between different atom types.

  Perturbs $\boldsymbol{h}_0$ to obtain $\boldsymbol{h}_{t_\tau}$:

  $\log c' = \log(\bar{\alpha}_{t_\tau}\boldsymbol{h}_0 + (1-\bar{\alpha}_{t_\tau})/K)$,

  $\boldsymbol{h}_{t_\tau} = \texttt{one\_hot}(\mathrm{argmax}_i[g_i + \log c_i])$, where $g \sim \mathrm{Gumbel}(0,1)$,

  Predicts $[\hat{\boldsymbol{x}}_0, \hat{\boldsymbol{f}}_0]$ from $[\boldsymbol{x}_{t_\gamma}, \boldsymbol{f}_{t_\gamma}]$ with ϕ_θ: $[\hat{\boldsymbol{x}}_0, \hat{\boldsymbol{f}}_0] = \phi_\theta([\boldsymbol{x}_{t_\gamma}, \boldsymbol{f}_{t_\gamma}], t, P)$.

  for $\boldsymbol{v}^i$ in $\{\mathcal{V}_{i=1}^{n}\}$ **do**

    Predicts $[\hat{\boldsymbol{v}}_0^i, \hat{\boldsymbol{h}}_0]$ from $[\boldsymbol{v}_{t_\tau}^i, \boldsymbol{h}_{t_\tau}]$ with ψ_θ: $[\hat{\boldsymbol{v}}_0^i, \hat{\boldsymbol{h}}_0] = \phi_\theta([\boldsymbol{v}_{t_\tau}^i, \boldsymbol{h}_{t_\tau}], t, P)$,

  Computes the posterior atom types $\boldsymbol{c}(\boldsymbol{f}_t, \boldsymbol{f}_0)$ and $\boldsymbol{c}(\boldsymbol{f}_{t_\gamma}, \hat{\boldsymbol{f}}_0)$.

  Computes the posterior atom types $\boldsymbol{c}(\boldsymbol{h}_t, \boldsymbol{h}_0)$ and $\boldsymbol{c}(\boldsymbol{h}_{t_\tau}, \hat{\boldsymbol{h}_0})$.

  Computes the unweighted MSE loss on atom coordinates, the KL loss on posterior atom types and weighted manifold-constrained loss: $L = \|\boldsymbol{x}_0 - \hat{\boldsymbol{x}_0}\|^2 + \mathrm{KL}(\boldsymbol{c}(\boldsymbol{f}_{t_\gamma}, \boldsymbol{f}_0) \parallel \boldsymbol{c}(\boldsymbol{f}_{t_\gamma}, \hat{\boldsymbol{f}}_0)) + \|\boldsymbol{v}_0 - \hat{\boldsymbol{v}_0}\|^2 + \mathrm{KL}(\boldsymbol{c}(\boldsymbol{h}_{t_\tau}, \boldsymbol{h}_0) \parallel \boldsymbol{c}(\boldsymbol{h}_{t_\tau}, \hat{\boldsymbol{h}}_0)) + \sum_{i=1}^{n} \|\|\hat{\boldsymbol{v}}_0^i - \hat{\boldsymbol{x}_0}\| - d_{\text{van der Waals radii}}\|$.

Algorithm 2 Sampling algorithm of NucleusDiff

Require: The protein binding site $\mathcal{P}$, the learned model ϕ_θ for modeling ligand distribution.

Samples the number of atoms in $\mathcal{M}$ based on a prior distribution conditioned on the pocket size.

Moves CoM of protein atoms to zero.

Samples initial molecular atom coordinates $\boldsymbol{x}_T$ and atom types $\boldsymbol{v}_T$:

$\boldsymbol{x}_T \in N(0, \boldsymbol{I})$

$\boldsymbol{f}_T = \texttt{one\_hot}(\mathrm{argmax}_i g_i)$, where $g \sim \mathrm{Gumbel}(0,1)$

for t in $T, T-1, \ldots, 1$ **do**

  Predicts $[\hat{\boldsymbol{x}_0}, \hat{\boldsymbol{f}}_0]$ from $[\boldsymbol{x}_t, \boldsymbol{f}_t]$ with ϕ_θ: $[\hat{\boldsymbol{x}_0}, \hat{\boldsymbol{f}}_0] = \phi_\theta([\boldsymbol{x}_t, \boldsymbol{f}_t], t, P)$

  Samples $\boldsymbol{x}_{t-1}$ from the posterior $p_\theta(\boldsymbol{x}_{t-1}|\boldsymbol{x}_t, \hat{\boldsymbol{x}_0})$

  Samples $\boldsymbol{f}_{t-1}$ from the posterior $p_\theta(\boldsymbol{f}_{t-1}|\boldsymbol{f}_t, \hat{\boldsymbol{f}}_0)$

---

5. Experiment Setup

In this section, we present our approach for constructing CrossDock datasets, describe our evaluation protocol, and provide comprehensive details on our algorithm as well as the baseline methods utilized in our study. To achieve this, we leverage sophisticated techniques and utilize advanced metrics to ensure the reliability and relevance of our results.

A. CrossDock Datasets.

CrossDock Datasets Construction. We conduct experiments to evaluate the generative performance of NucleusDiff on the CrossDocked2020 dataset (25). This dataset comprises 22.5 million docked protein-ligand pairs, with each pair exhibiting various poses across multiple pockets within the Protein Data Bank. The ligands associated with specific pockets were docked with each receptor assigned to those pockets using smina through Pocketome. Binding data (pK) for the CrossDocked2020 set were sourced from PDBbind v2017, revealing that 41.9% of the complexes have available binding affinity data. For a fair comparison, we follow previous works (26, 27) by selecting only binding pose data with root-mean-squared deviations (RMSD) of less than 1 Å . we further refine the dataset through clustering at 30% sequence identity using `MMseqs2` (28). This process yields 100,000 pairs for training and 100 pairs for evaluation.

CrossDock Mesh Datasets Construction. We utilize `MSMS` (29) to compute the solvent-excluded surface of the molecule, employing a probe radius of 1.5 Å and a sampling density of 3.0 for small molecules, generating a triangular mesh representation. To further refine the surface mesh, we employ `PyMesh` (30), which helps in reducing the number of vertices and correcting poorly meshed regions. Addressing degenerate vertices or disconnected surfaces is crucial, as these issues can lead to an improper distribution of mesh points when training the models. Finally, we selected the K mesh points that are closest to the van der Waals radii distance from the nucleus to construct a mesh point dataset for the ligand. This dataset predominantly includes the 3D coordinates of the mesh points.

B. Training Details. The two score model are trained using the gradient descent method Adam (31) with `init_learning_rate=0.001`, `betas=(0.95, 0.999)`, `batch_size=4`, and `clip_gradient_norm=8`. To balance the scales of the two losses, we apply a factor of $\alpha = 100$ to the atom type loss. During the training phase, we add small Gaussian noise with a standard deviation of 0.1 to protein atom coordinates as data augmentation. We also schedule to decay the learning rate exponentially with `a factor of 0.6` and `a minimum learning rate of 1e-6`. The learning rate is decayed if there is no improvement in the validation loss over `10 consecutive evaluations`. The evaluation is performed for `every 100 training steps`.

C. Implementation Details. Our NucleusDiff comprises two score models, each consisting of 9 equivariant layers. Each layer is a Transformer (32) with `hidden_dim=128` and `n_heads=16`. The key/value embeddings and attention scores are generated through a 2-layer MLP with LayerNorm and ReLU activation. For atom coordinates, we use a sigmoid β schedule with $\beta_1 =$ `1e-7` and $\beta_T =$ `2e-3`. For atom types, we adopt a cosine β schedule as suggested by (33), with `s=0.01`. We set the number of diffusion timesteps to `1000`.

D. Baselines. We conducted a comparative evaluation of NucleusDiff with leading generative models for structure-based drug design, including liGAN [*], GraphBP [†], AR-SBDD [‡], Pocket2Mol [§], and TargetDiff [¶]. For each comparison model, we utilized the source code obtained from the respective repositories.

E. Configuration. All algorithms and models are developed using Python 3.8.13, with PyTorch version 1.12.1 and PyTorch Geometric version 2.5.2, under CUDA 11.0. Experiments are conducted on a server with 8 NVIDIA V100 GPUs (32 GB memory) and Intel(R) Xeon(R) Platinum 8255C CPU @ 2.50GHz. We employ a single V100 GPU for model training and leverage eight GPUs to accelerate the sampling procedure.

[*] LiGAN (GPL-2.0 license): https://github.com/mattragoza/LiGAN.
[†] GraphBP (GPL-3.0 license): https://github.com/divelab/GraphBP.
[‡] AR-SBDD (MIT license): https://github.com/luost26/3D-Generative-SBDD.
[§] Pocket2Mol (MIT license): https://github.com/pengxingang/Pocket2Mol.
[¶] TargetDiff (MIT license): https://github.com/guanjq/targetdiff.

6. More Experiment Results

In this section, we conduct additional experiments to further assess the effectiveness of our proposed model, particularly in addressing the *atomic collision issue*.

A. More Results on Evaluating Atomic Collision Issues. Table S1 presents a comprehensive evaluation of *collision issues* (PLCR, ALCR, MLCR) from Step-0 to Step-1000 during the inference phase. The experimental results suggest that NucleusDiff and TargetDiff have similar performance concerning collision issues in the early stages of inference (Step-0 to Step-300). However, from Step-400 onward, NucleusDiff demonstrates a significantly faster convergence rate in addressing atomic collision problems. By approximately Step-700, NucleusDiff appears to almost completely resolve the collision issues. In contrast, TargetDiff shows rapid convergence in addressing collision problems from Step-400 to Step-600, after which its performance related to collision issues shows little to no change. Table S1 offers a comprehensive overview of how these two diffusion-based models handle collision issues during the inference phase, giving us a clearer understanding of how the pretrained NucleusDiff model mitigates such problems.

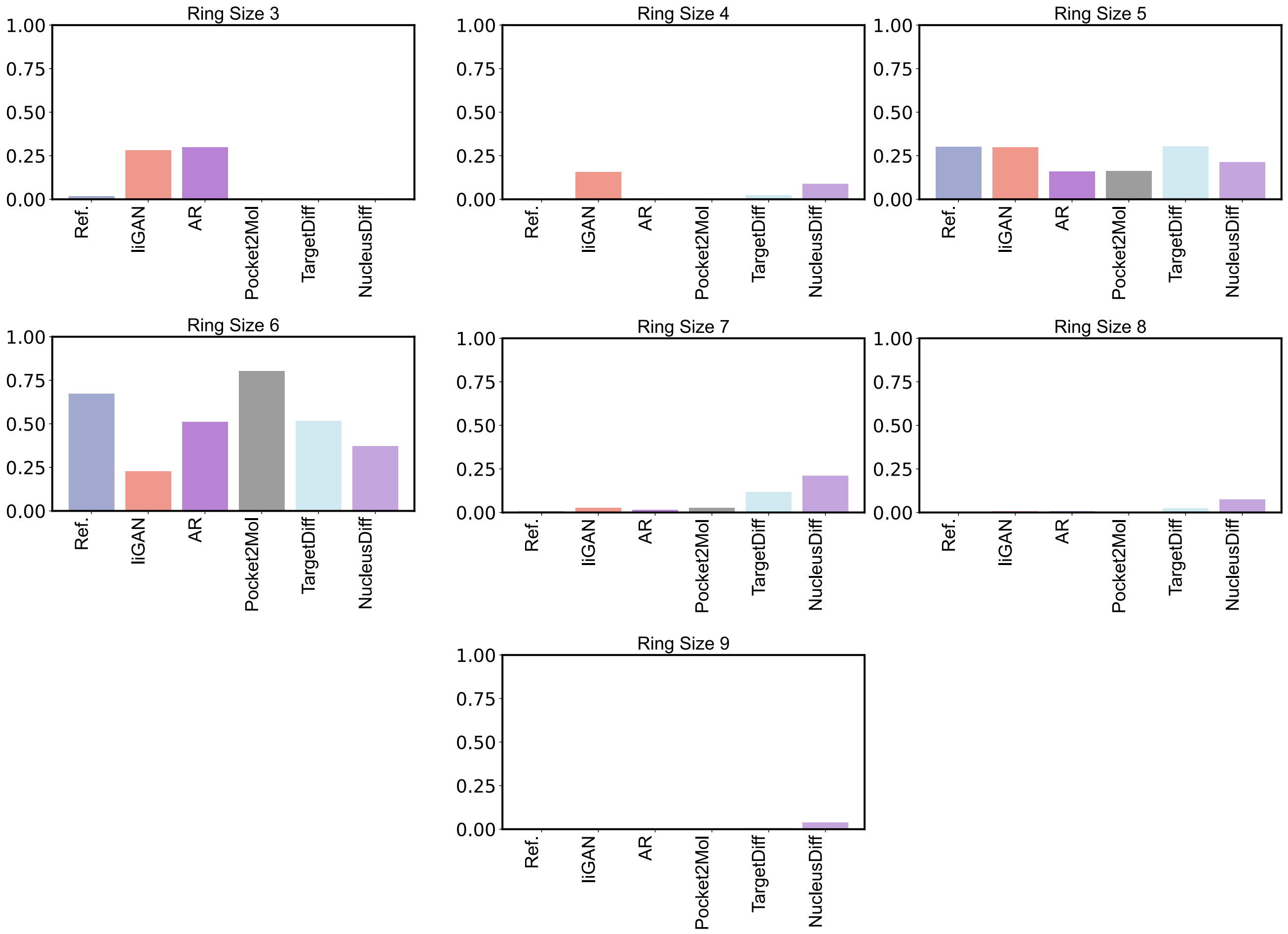

Fig. S2. The ring size distribution of molecules generated by the baseline models and NucleusDiff.

B. The Ring Size distribution. Many previous studies (9, 26, 27) have suggested that if the ligands generated by a pretrained model exhibit high structural consistency with the ground-truth ligands in the test set, the generative model can be considered highly successful. However, we argue that this perspective is flawed. A generative model should not only learn a distribution but also possess strong generalization capabilities. Our goal is to generate a diverse array of ligands beyond the training distribution, which holds significant practical implications for drug design and discovery. In this section, we present a detailed analysis of the substructures of the molecules generated by NucleusDiff, specifically focusing on the distribution of ring sizes in the generated molecules. Figure S2 illustrates the distribution of different ring sizes present in the test set, as well as in

Table S1. The atomic collision performance among pocket-ligand pairs for structure-based drug design. A lower value is better.

Metrics	TargetDiff			NucleusDiff (ours)		
	PLCR	ALCR	MLCR	PLCR	ALCR	MLCR
**Step-0**	17103/2300930	13241/230093	4425/10000	17120/2300930	13257/230093	4428/10000
**Step-100**	11293/2300930	9083/230093	3613/10000	11185/2300930	9040/230093	3627/10000
**Step-200**	6019/2300930	5079/230093	2543/10000	5779/2300930	4918/230093	2540/10000
**Step-300**	2482/2300930	2142/230093	1285/10000	2081/2300930	1847/230093	1254/10000
**Step-400**	751/2300930	671/230093	426/10000	444/2300930	414/230093	321/10000
**Step-500**	211/2300930	183/230093	124/10000	90/2300930	80/230093	64/10000
**Step-600**	84/2300930	77/230093	56/10000	29/2300930	28/230093	26/10000
**Step-700**	78/2300930	70/230093	45/10000	7/2300930	7/230093	7/10000
**Step-800**	77/2300930	70/230093	45/10000	4/2300930	4/230093	4/10000
**Step-900**	78/2300930	70/230093	43/10000	2/2300930	2/230093	2/10000
**Step-1000**	65/2300930	60/230093	37/10000	0/2300930	0/230093	0/10000

the 10,000 molecules generated by the baselines and NucleusDiff. The results reveal that, in comparison to the test set and another diffusion-based model, TargetDiff, the ring sizes in the molecules generated by these models are primarily concentrated around 5 and 6. In contrast, the ring sizes in the molecules generated by NucleusDiff are mainly distributed among 5, 6, 7, 8, and 9. Notably, the proportion of ring sizes 7, 8, and 9 is significantly higher in NucleusDiff compared to TargetDiff and the test set. This observation leads to two key insights: (1) NucleusDiff demonstrates the potential to generate more complex structures, such as intricate ring structures, compared to TargetDiff. (2) The structures generated by NucleusDiff are more novel, as evidenced by the discrepancy between the substructure distribution of the ground-truth ligands in the test set and that of the molecules generated by NucleusDiff.

Table S2. The distribution of ring sizes in the test set and molecules generated by the models.

Ring Size	Ref.	liGAN	AR	Pocket2Mol	TargetDiff	NucleusDiff (ours)
**3**	1.7%	28.1%	29.9%	0.1%	0.0%	0.0%
**4**	0.0%	15.7%	0.0%	0.0%	2.5%	8.8%
**5**	30.2%	29.8%	16.0%	16.4%	30.6%	21.4%
**6**	67.4%	22.7%	51.2%	80.4%	51.8%	37.3%
**7**	0.7%	2.6%	1.7%	2.6%	11.8%	21.2%
**8**	0.0%	0.8%	0.7%	0.3%	2.5%	7.6%
**9**	0.0%	0.3%	0.5%	0.1%	0.8%	3.7%

C. The Bond Distribution. In our study, we evaluate the performance of various generative models in terms of their ability to reproduce the bond distributions observed in reference molecules. Our primary focus was on the NucleusDiff model, which demonstrated a unique capability in generating diverse molecular substructures.

Figure S3 presents a comparative analysis of the bond distributions for different models, including liGAN, GraphBP, AR, Pocket2Mol, TargetDiff, and NucleusDiff. The NucleusDiff model consistently shows a balanced distribution across various bond types (C-C, C=C, C-N, C=N, C-O, C=O, C:C, C:N), indicating its proficiency in capturing the structural diversity inherent in the reference dataset.

Table S3 provides quantitative insights through the Jensen-Shannon divergence between the bond distance distributions of reference molecules and those generated by each model. The NucleusDiff model exhibits competitive divergence values across all bond types, highlighting its effectiveness in mimicking the bond length distributions of real molecules. The Jensen-Shannon divergence is a measure of similarity between two probability distributions, with values ranging from 0 to 1. Lower values indicate higher similarity between distributions. In this context, the low divergence values achieved by NucleusDiff, particularly for bonds like C=N (0.649) and C=O (0.464), signify that the bond length distributions in the generated molecules closely resemble those in the reference molecules. This high degree of similarity suggests that NucleusDiff can accurately capture and reproduce the structural characteristics of real molecules. Notably, for bonds like C=N and C=O, NucleusDiff achieves divergence values of 0.649 and 0.464, respectively, which are relatively low and suggest a high degree of similarity to the reference distributions.

The superior performance of NucleusDiff in generating diverse substructures can be attributed to its advanced architectural design, which allows for fine-grained control over molecular features. While other models like liGAN and GraphBP show competence in certain aspects of molecular generation, they often struggle with maintaining consistent performance across various bond types. For instance, liGAN exhibits higher divergence values for most bond types, indicating less accurate reproduction of bond distributions. GraphBP, while performing well for some bonds, shows inconsistency across different bond types. In contrast, NucleusDiff demonstrates a more balanced and consistently low divergence across all bond types,

highlighting its superior capability in generating diverse and structurally accurate molecules. This is evident from its ability to generate molecules with a wide range of bond types, maintaining a high degree of structural fidelity to natural molecules. Consequently, NucleusDiff not only ensures the generation of chemically valid molecules but also enhances the exploration of the chemical space by producing a variety of substructures, which is crucial for applications in drug discovery and materials science.

In summary, the NucleusDiff model stands out in its ability to generate molecular substructures with considerable diversity, closely mirroring the bond distributions of reference molecules. This capability underscores its potential as a powerful tool for the generation of novel and diverse molecular entities.

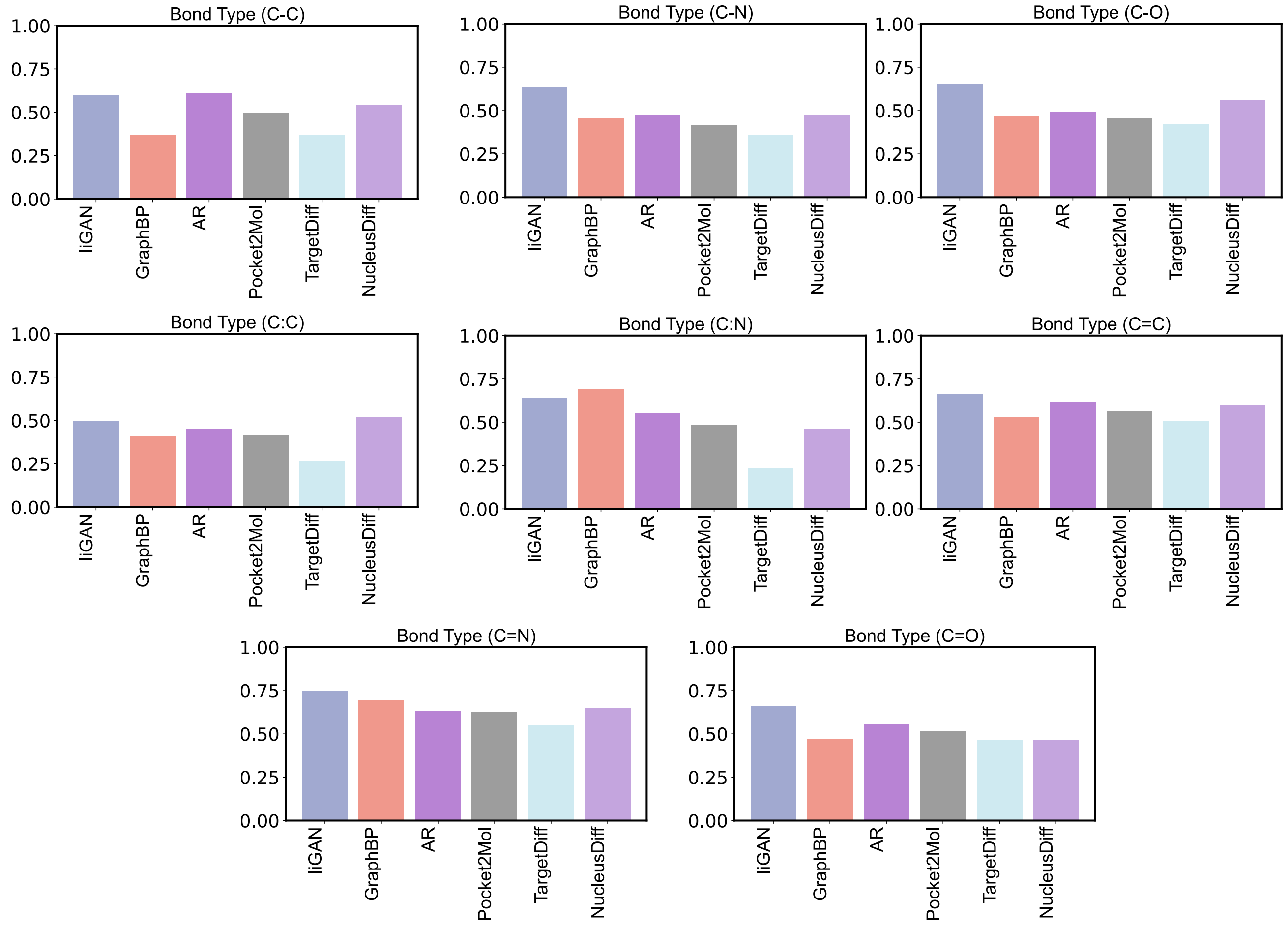

Fig. S3. The bond distribution of the molecules generated by baseline models and NucleusDiff.

Table S3. The Jensen-Shannon divergence between the distributions of bond distances for reference versus generated molecules is analyzed. In this context, "-", "=", and ":" denote single, double, and aromatic bonds, respectively.

Bond	liGAN	GraphBP	AR	Pocket2Mol	TargetDiff	NucleusDiff (ours)
**C−C**	0.601	0.368	0.609	0.496	0.367	0.544
**C=C**	0.665	0.530	0.620	0.561	0.507	0.599
**C−N**	0.634	0.456	0.474	0.416	0.361	0.478
**C=N**	0.749	0.693	0.635	0.629	0.551	0.649
**C−O**	0.656	0.467	0.492	0.454	0.424	0.559
**C=O**	0.661	0.471	0.558	0.516	0.467	0.464
**C:C**	0.497	0.407	0.451	0.416	0.264	0.517
**C:N**	0.638	0.689	0.552	0.487	0.234	0.464

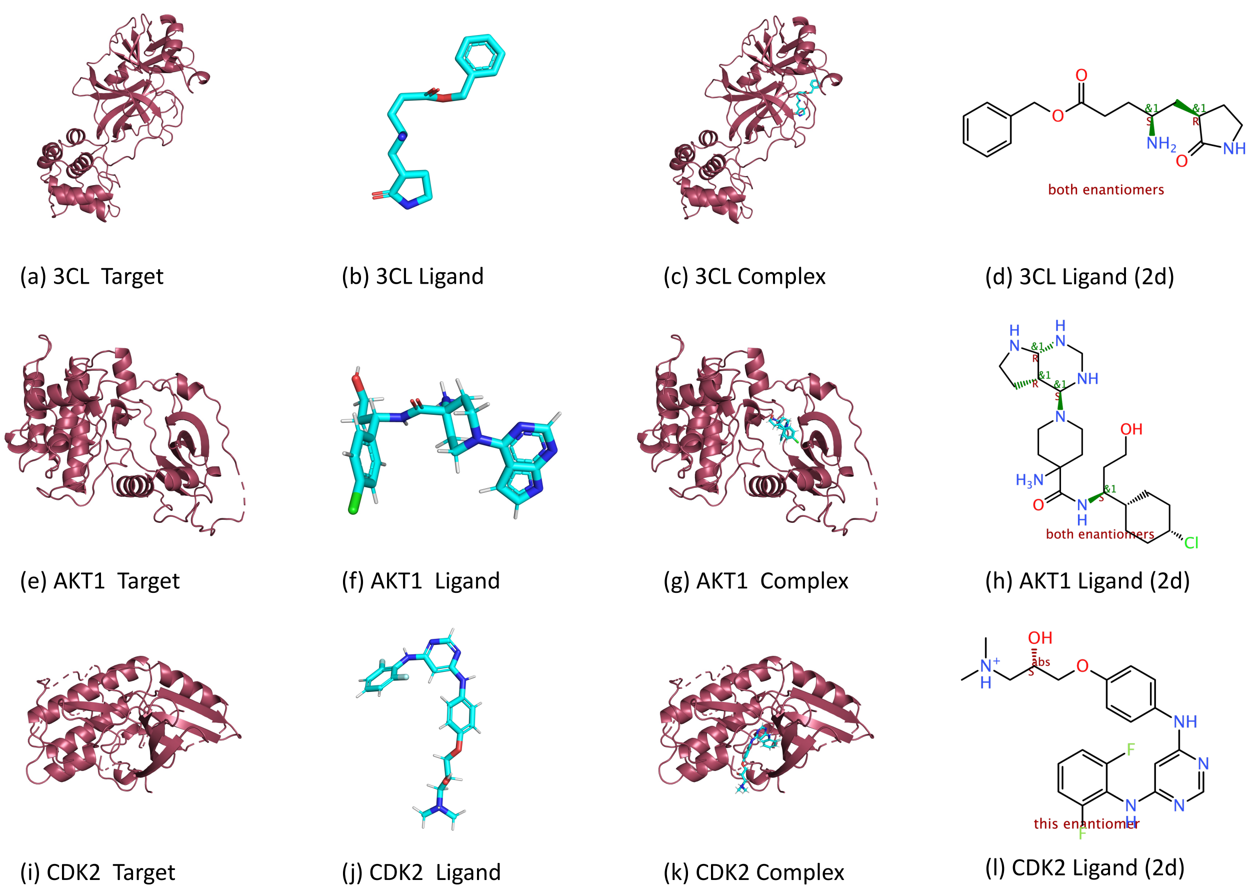

Fig. S4. Visualization for drug design on COVID-19 and the other two therapeutic targets.

D. More Experiments for Advances in Drug Design for COVID-19 and Other Therapeutic Targets. While evaluating a model's performance on representative test sets is a common practice in the machine learning community, scientists and drug designers are more interested in the models' performance in real-world applications. Therefore, following prior work (16), we conduct more experiments on a carefully curated dataset comprising over three out-of-distribution therapeutic targets, including those related to COVID-19, along with their experimentally validated active ligands. This dataset is designed to more accurately reflect the models' potential in practical settings. In this context, we generate molecules targeting these specific therapeutic targets and subsequently evaluate their binding affinity, drug-likeness properties, and similarity to known active compounds. Additionally, we assess the performance of NucleusDiff and TargetDiff on the three therapeutic targets (including the COVID-19 target) using the proposed collision metrics. This step is critical for comprehensively analyzing whether NucleusDiff can effectively address collision issues in structure-based drug design for real-world therapeutic targets, which is a central focus of our research.

Experimental Setup. Here, we employ the pretrained models of TargetDiff and NucleusDiff to sample 1000 ligands for each target: **3CL**, **ATK1**, and **CDK2**. Both models utilize diffusion-based methodologies, facilitating the generation of molecular structures through iterative refinements. For each target, the sampling process is conducted over 1000 timesteps to ensure the convergence of molecular structures. The generated ligands are evaluated using several metrics to comprehensively assess their performance. Specifically, we use AutoDock Vina to compute binding affinity metrics, including the Vina Score, Vina Min, and Vina Dock. The Vina Score measures the overall binding affinity, while Vina Min and Vina Dock provide insights into the minimum and docking scores, respectively, indicating the strength of ligand-target interactions. High Affinity metrics are also calculated to gauge the proportion of generated ligands with exceptionally strong binding affinity. Drug-likeness properties are assessed using the Quantitative Estimate of Drug-likeness (QED) score, which evaluates the likelihood of a compound to exhibit drug-like characteristics. Additionally, we measure synthetic accessibility (SA), which quantifies the ease with which a compound can be synthesized, and molecular diversity, which assesses the range and heterogeneity of structures within the generated ligand set. To further analyze the models' performance in addressing atomic collisions, we examine atomic collision ratios at 11 timesteps, sampled every 100 timesteps from Step-0 to Step-1000. The metrics used for this analysis included PLCR, ALCR, and MLCR. These metrics provide insights into how the atomic collision issue evolved during the inference process, with lower values indicating fewer collisions and better structural compatibility. This comprehensive experimental setup allows

Table S4. The atomic collision performance among pocket-ligand pairs for structure-based drug design in the three real-world therapeutic targets. A lower value is better.

**Metrics**	**TargetDiff**			**NucleusDiff (ours)**		
	**PLCR**	**ALCR**	**MLCR**	**PLCR**	**ALCR**	**MLCR**
**Step-0**	15931/810000	10979/81000	1496/3000	15924/810000	10973/81000	1499/3000
**Step-100**	10327/810000	7490/81000	1329/3000	9369/810000	6673/81000	1328/3000
**Step-200**	5611/810000	4125/81000	1147/3000	4329/810000	3556/81000	1158/3000
**Step-300**	2258/810000	1695/81000	779/3000	1345/810000	1151/81000	716/3000
**Step-400**	719/810000	547/81000	330/3000	285/810000	241/81000	212/3000
**Step-500**	166/810000	127/81000	83/3000	52/810000	46/81000	41/3000
**Step-600**	42/810000	36/81000	26/3000	14/810000	12/81000	11/3000
**Step-700**	23/810000	20/81000	17/3000	5/810000	4/81000	4/3000
**Step-800**	17/810000	16/81000	12/3000	1/810000	1/81000	1/3000
**Step-900**	12/810000	10/81000	8/3000	1/810000	1/81000	1/3000
**Step-1000**	5/810000	5/81000	3/3000	3/810000	3/81000	3/3000

Table S5. A summary of 14 biochemical properties for molecules generated by TargetDiff and NucleusDiff for target 3CL. The symbols (↑) and (↓) indicate whether a higher or lower value is preferable for each property.

**Metrics**	**Vina Score (↓)**		**Vina Min (↓)**		**Vina Dock (↓)**		**High Affinity (↑)**		**QED (↑)**		**SA (↑)**		**Diversity (↑)**	
	**Avg.**	**Med.**	**Avg.**	**Med.**	**Avg.**	**Med.**	**Avg.**	**Med.**	**Avg.**	**Med.**	**Avg.**	**Med.**	**Avg.**	**Med.**
**TargetDiff**	-4.82	-5.08	-5.61	-5.68	-6.39	-6.49	50.5%	50.5%	**0.56**	**0.54**	**0.62**	**0.61**	**0.76**	**0.76**
**NucleusDiff (ours)**	**-5.85**	**-5.80**	**-6.21**	**-6.23**	**-6.74**	**-6.84**	**70.0%**	**70.0%**	0.43	0.42	0.54	0.53	0.73	0.73

Table S6. A summary of 14 biochemical properties for molecules generated by TargetDiff and NucleusDiff for target AKT1. The symbols (↑) and (↓) indicate whether a higher or lower value is preferable for each property.

**Metrics**	**Vina Score (↓)**		**Vina Min (↓)**		**Vina Dock (↓)**		**High Affinity (↑)**		**QED (↑)**		**SA (↑)**		**Diversity (↑)**	
	**Avg.**	**Med.**	**Avg.**	**Med.**	**Avg.**	**Med.**	**Avg.**	**Med.**	**Avg.**	**Med.**	**Avg.**	**Med.**	**Avg.**	**Med.**
**TargetDiff**	-8.31	-8.22	-8.77	-8.69	-9.24	-9.17	34.2%	34.2%	**0.43**	**0.42**	**0.51**	**0.52**	0.55	0.55
**NucleusDiff (ours)**	**-9.42**	**-9.37**	**-9.33**	**-9.39**	**-9.84**	**-9.80**	**52.8%**	**52.8%**	0.29	0.27	0.41	0.43	**0.56**	**0.56**

for a rigorous evaluation of NucleusDiff and TargetDiff, highlighting their performance in generating high-quality ligands for the specified therapeutic targets. The results, detailed in the subsequent sections and tables, underscore the strengths of each model and identify potential areas for improvement in the context of structure-based drug design.

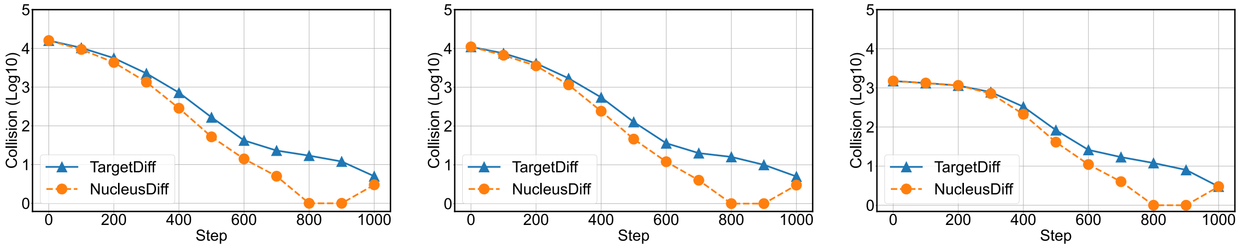

(a) Performance of PLCR (b) Performance of ALCR (c) Performance of MLCR

Fig. S5. Visualization of the atomic collision ratios for TargetDiff and NucleusDiff across three targets (3CL, AKT1, CDK2).

Atomic Collision Evaluation. To evaluate the performance of NucleusDiff, we conduct a comprehensive comparison with TargetDiff, focusing on their application to real-world scenarios, including therapeutic targets such as COVID-19. Both methods leverage diffusion-based models, which enable a thorough understanding of the atomic collision issue during the inference process of DDPM (34). Our datasets include three real-world therapeutic targets (3CL, AKT1, CDK2) with experimentally validated active ligands. We analyze three key metrics for atomic collision at 11 timesteps, sampled every 100 timtsteps from Step-0 to Step-1000. The main results are summarized in Table S4, which presents the collision metrics for each method across these steps. In Table S4, we observe that TargetDiff shows a significant reduction in atomic collisions from Step-0 to Step-1000. Conversely,

Table S7. A summary of 14 biochemical properties for molecules generated by TargetDiff and NucleusDiff for target CDK2. The symbols (↑) and (↓) indicate whether a higher or lower value is preferable for each property.

Metrics	Vina Score (↓)		Vina Min (↓)		Vina Dock (↓)		High Affinity (↑)		QED (↑)		SA (↑)		Diversity (↑)	
	Avg.	Med.	Avg.	Med.	Avg.	Med.	Avg.	Med.	Avg.	Med.	Avg.	Med.	Avg.	Med.
TargetDiff	-8.96	-8.93	-9.43	-9.39	-9.91	-9.87	**97.0%**	**97.0%**	**0.52**	**0.52**	**0.50**	**0.51**	0.55	0.55
NucleusDiff (ours)	**-10.78**	**-11.02**	**-10.71**	**-11.10**	**-11.10**	**-11.30**	95.7%	95.7%	0.38	0.38	0.38	0.36	**0.59**	**0.59**

NucleusDiff maintains a consistently lower collision ratio over the same inference steps. Notably, NucleusDiff significantly outperforms TargetDiff across all three collision metrics (PLCR, ALCR, and MLCR), with the difference approaching an order of magnitude. Specifically, in the final sampling steps, NucleusDiff achieves an almost negligible collision ratio, underscoring its superior performance. Referring to Figure S5, which visualizes the atomic collision ratio for both methods across three targets (3CL, AKT1, CDK2), we can see a marked contrast in the convergence trends. NucleusDiff demonstrates a more pronounced and rapid reduction in collision ratios compared to TargetDiff. This suggests that NucleusDiff is better suited to real-world applications, especially for real-world therapeutic targets. The enhanced convergence and lower collision ratios of NucleusDiff highlight its potential for practical deployment in drug design.

Binding Affinity Evaluation. For each target protein, we generate 1000 ligand molecules, resulting in a total of 3000 molecules per model. The comprehensive results for NucleusDiff and TargetDiff are displayed in Table S5, Table S6, and Table S7. We note that NucleusDiff outperforms TargetDiff in 8 out of the 14 evaluated metrics across all three therapeutic targets. According to the Vina Score, NucleusDiff achieves an average score of -5.85 for 3CL, -9.42 for AKT1, and -10.78 for CDK2, indicating superior binding affinity compared to TargetDiff. Similarly, for the Vina Min and Vina Dock metrics, NucleusDiff consistently outperforms TargetDiff, suggesting that NucleusDiff is more effective in predicting highly favorable binding poses. In terms of High Affinity, NucleusDiff exhibits a significant advantage over TargetDiff, with 70.0% for 3CL, 52.8% for AKT1, and 95.7% for CDK2, compared to 50.5%, 34.2%, and 97.0% respectively for TargetDiff. This indicates that NucleusDiff is more proficient at generating ligands with strong binding interactions. Regarding the QED metric, although NucleusDiff's scores are slightly lower than those of TargetDiff, it still maintains acceptable drug-likeness properties. For instance, the QED scores for NucleusDiff are 0.43 for 3CL, 0.29 for AKT1, and 0.38 for CDK2, compared to TargetDiff's 0.56, 0.43, and 0.52 respectively. The SA scores for NucleusDiff are within a reasonable range, ensuring that the generated molecules are synthetically accessible. The Diversity metric further underscores the capability of NucleusDiff to explore a broader chemical space, with scores of 0.73, 0.56, and 0.59 for 3CL, AKT1, and CDK2 targets respectively, compared to TargetDiff's 0.76, 0.55, and 0.55. These results collectively demonstrate that NucleusDiff not only excels in generating high-affinity ligands but also maintains a balance between drug-likeness, synthetic accessibility, and structural diversity. Consequently, NucleusDiff shows great potential for generating viable drug candidates in real-world therapeutic settings, including those for COVID-19.

Visual Analysis of NucleusDiff. Figure S6, Figure S7, and Figure S8 illustrate the visual representation of the ligands generated by NucleusDiff and TargetDiff for several therapeutic targets, including COVID-19 targets such as 3CL, AKT1, and CDK2. We select these targets due to their significance in current drug design efforts and to evaluate the robustness of the models in generating viable drug candidates. We observe that both TargetDiff and NucleusDiff demonstrate the potential to generate stable and biologically relevant structures. From the perspective of binding affinity, as indicated by the Vina Scores, NucleusDiff tends to produce ligands with higher affinity compared to those generated by TargetDiff. This trend is specifically evident in the ligands generated for the targets 3CL, AKT1, and CDK2, as shown in Figure S6, Figure S7, and Figure S8. When considering atomic collision in the generated molecules, a more detailed comparison between TargetDiff and NucleusDiff can be made. For instance, with the 3CL target, it is evident that the ligands generated by TargetDiff exhibit a less defined positioning relative to the binding pocket, leading to potential atomic collisions and suboptimal binding interactions. In contrast, the ligands generated by NucleusDiff show a much clearer boundary and more precise alignment with the protein pockets. This indicates that NucleusDiff learns both the relative positioning of ligands in protein pockets and the physical rules governing atomic and electronic distributions within ligands. Moreover, NucleusDiff's ability to generate ligands with higher binding affinities and better structural compatibility suggests its superior performance in real-world drug design scenarios. This is particularly crucial for rapidly evolving therapeutic targets such as those related to COVID-19, where the accuracy and efficiency of ligand generation can significantly impact the speed of drug development. In conclusion, the visual and quantitative analyses confirm that NucleusDiff outperforms TargetDiff in generating ligands with higher affinity and better structural compatibility for critical therapeutic targets. This enhanced performance underscores the potential of NucleusDiff in facilitating more effective and rapid drug discovery processes.

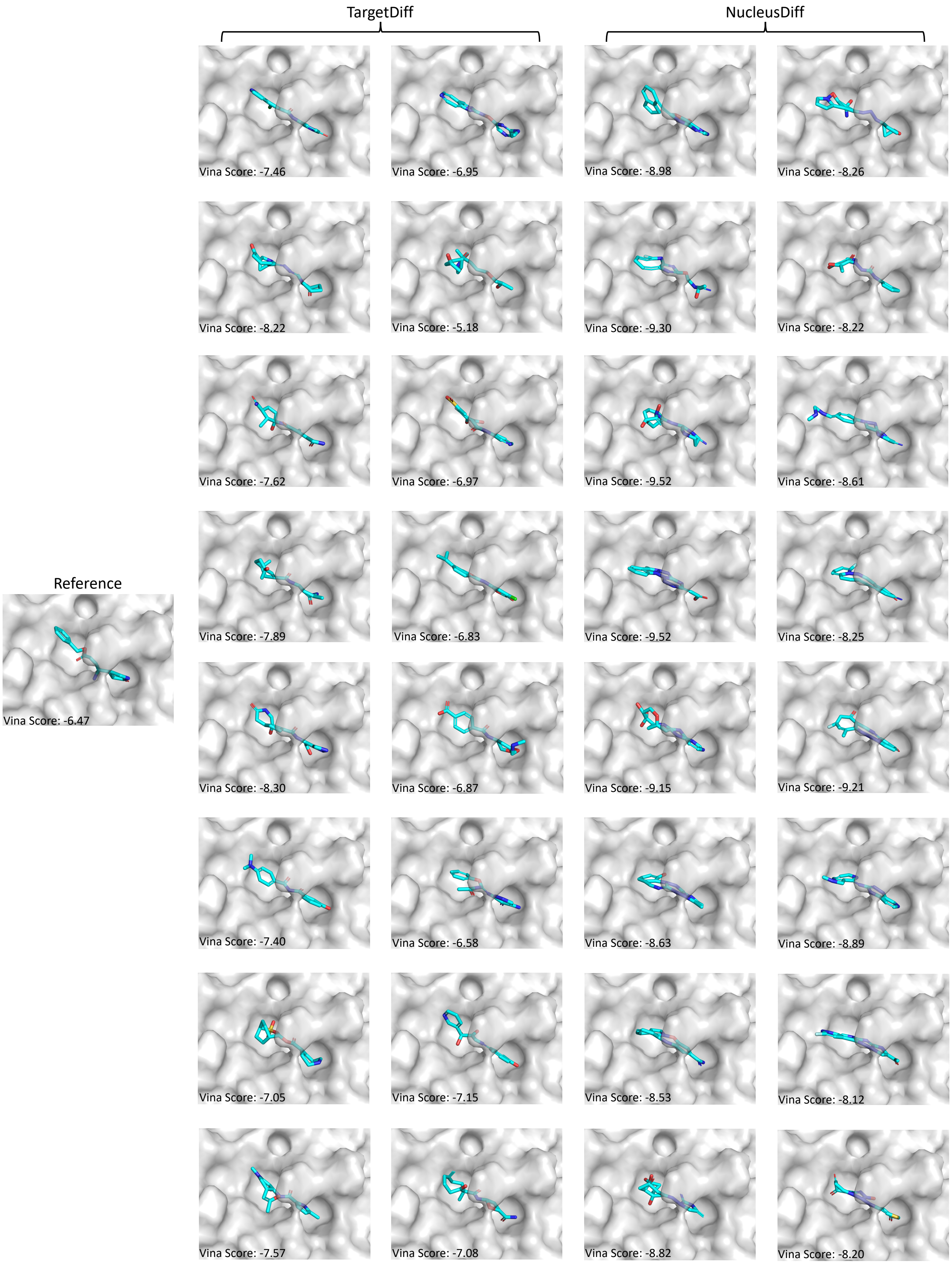

Fig. S6. Visualization of the generated molecules by TargetDiff and NucleusDiff for **target 3CL**.

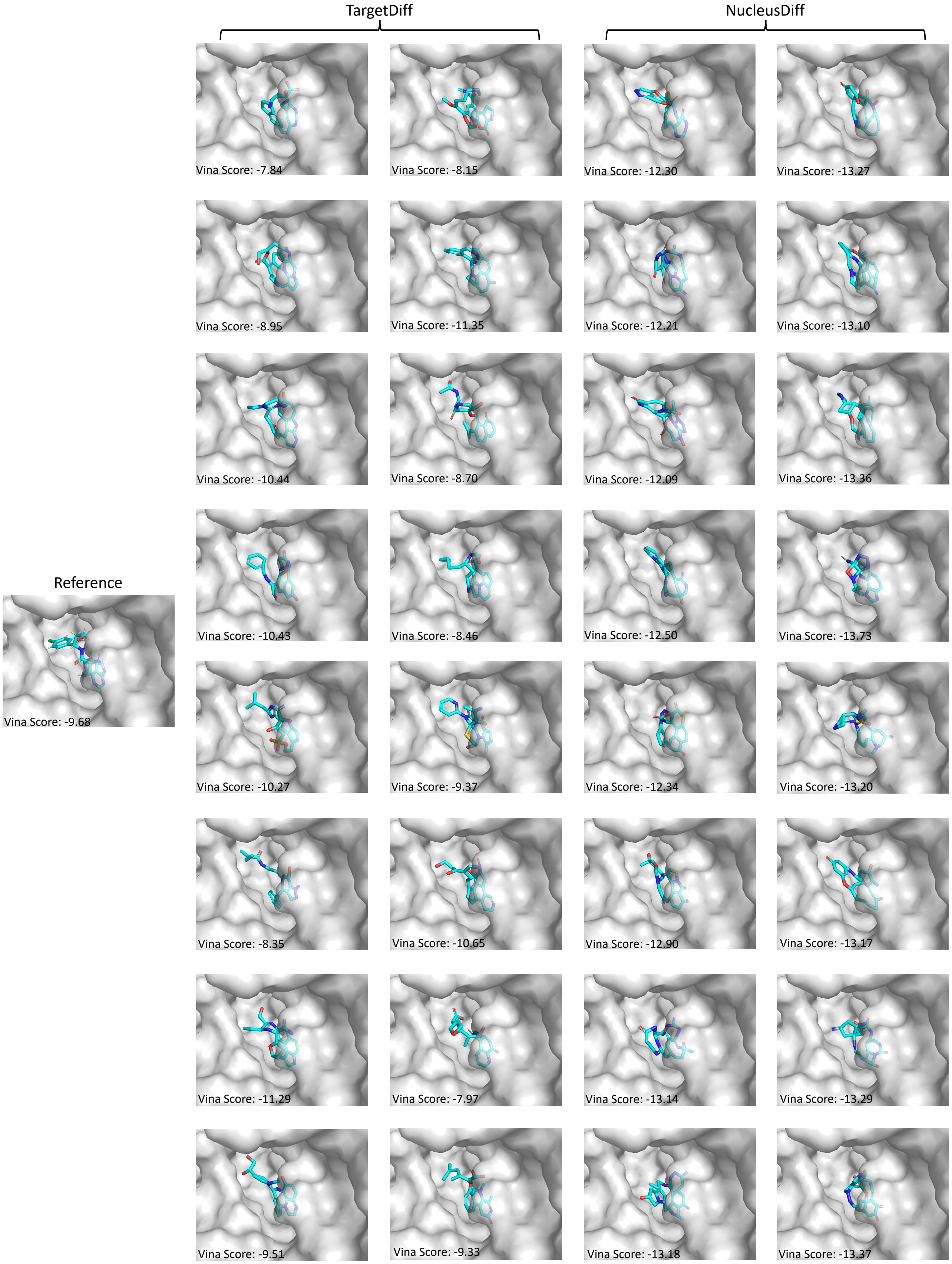

Fig. S7. Visualization of the generated molecules by TargetDiff and NucleusDiff for **target AKT1**.

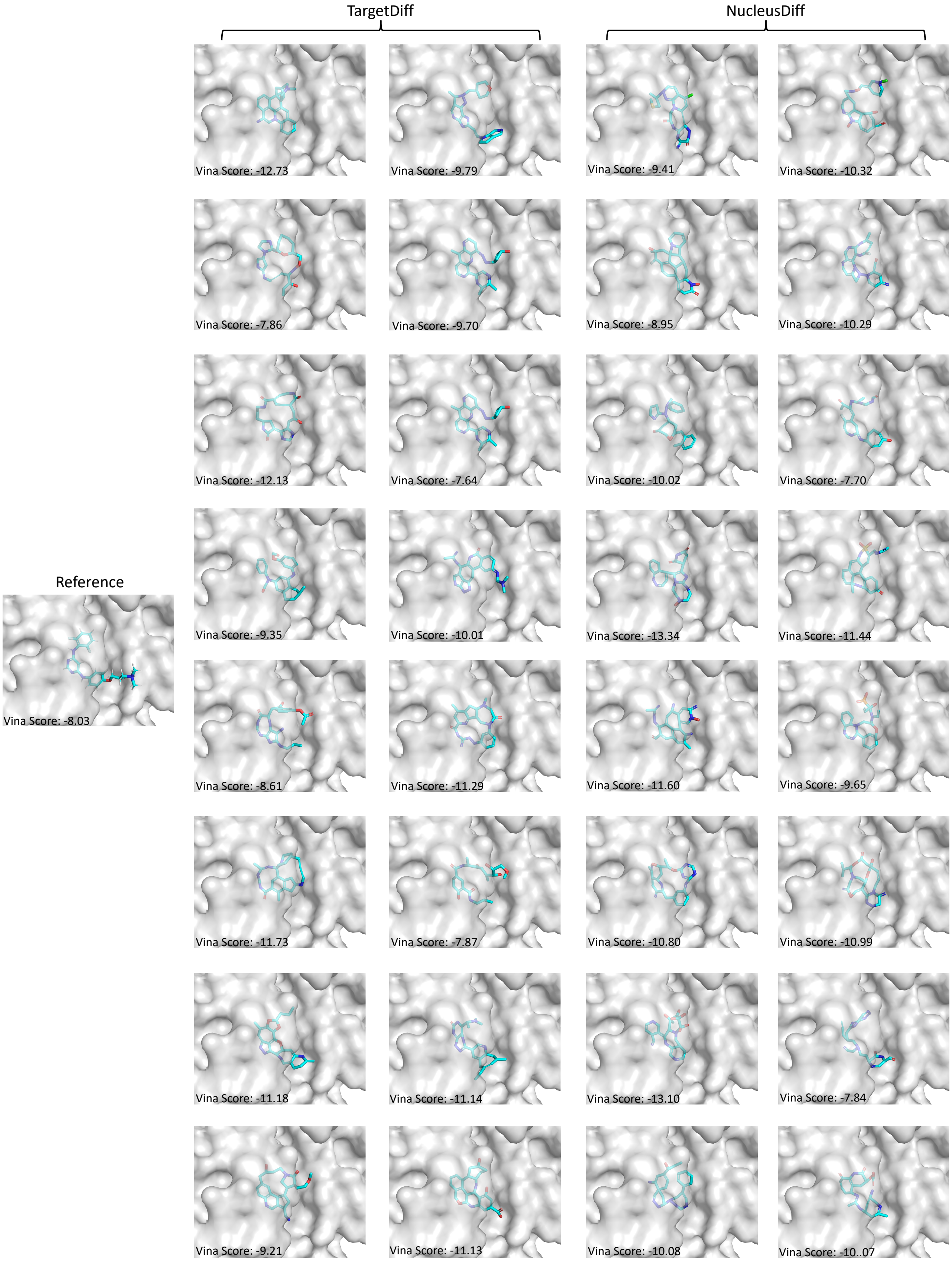

Fig. S8. Visualization of the generated molecules by TargetDiff and NucleusDiff for **target CDK2**.

E. More Visualization Results. Here, we visualize additional molecules generated by NucleusDiff and employ TargetDiff as a comparative framework to illustrate the spatial configurations of the synthesized molecules. Figure S9 presents additional visualizations of ligands generated by TargetDiff and NucleusDiff, compared against the reference ligands for several protein pockets. The selected protein pockets for this analysis include 14GS, 5W2G, 2RHY, 1GGS, 5TGN, 1DJY, 4F1M, 4TQR, 5NGZ, and 1K9T. The Vina scores demonstrate that NucleusDiff consistently generates ligands with higher binding affinities compared to both TargetDiff and the reference ligands across all analyzed protein pockets. We can group our findings as follows:

- For protein pockets 14GS, 5W2G, 2RHY, and 1GGS, NucleusDiff achieves significantly lower Vina scores, indicating stronger binding affinities. Specifically, NucleusDiff's scores for these pockets are -10.67, -9.32, -7.59, and -8.80 respectively, compared to TargetDiff's scores of -6.24, -5.85, -3.67, and -7.26.
- Similarly, for protein pockets 5TGN, 1DJY, 4F1M, 4TQR, 5NGZ, and 1K9T, NucleusDiff demonstrates superior performance in terms of binding affinity, consistently achieving lower Vina scores compared to TargetDiff and the reference ligands.

In addition to improved Vina scores, NucleusDiff also exhibits superior accuracy in the positional relationship between the ligands and the protein pockets, reducing the risk of atomic collision. This advantage is particularly evident in the following cases:

- For protein pockets 1DJY, 4F1M, and 4TQR, the ligands generated by NucleusDiff are more accurately positioned within the pockets, with clearer boundaries and better adherence to physical atomic interaction rules.
- In contrast, TargetDiff's generated ligands often exhibit less clear positioning and potential atomic collisions across various protein pockets.

The enhanced performance of NucleusDiff in both binding affinity and positional accuracy underscores its potential advantages in drug design and the modeling of protein-ligand interactions. These results highlight NucleusDiff's ability to generate stable and precisely positioned ligands, making it a more effective tool for computational drug discovery and optimization processes.

F. Visualization of the Training Process. The visualizations in Figure S10 provide insights into the training dynamics of the model. The Total Training Loss Curve (a) shows overall loss with noticeable fluctuations but a general downward trend, indicating that the model learns over time. The training loss curves for molecular and mesh modeling (b-f) exhibit significant variability, suggesting challenges in these specific tasks. In contrast, the Training Loss Curve for Mesh Feature (g) displays lower values and less fluctuation, indicating that the model finds it easier to learn mesh features. The Constrained Loss curve (h) shows fewer oscillations, implying that constraints help stabilize the training process. The Learning Rate Curve (i) remains relatively constant, suggesting a stable learning rate policy. The Gradient Normalization Curve (j) with occasional spikes indicates moments of large gradient changes, while the Atom Type Accuracy Curve (k) shows stable accuracy, reflecting consistent performance in predicting atom types. The Iteration Curve (l) linearly increases, reflecting the progression of training steps over time. The validation loss curves (m-o) mirror the training loss curves, showing high variability but general downward trends, which suggests that the model generalizes well to the validation data. Despite the fluctuations, the general improvement over time indicates effective learning. The stable learning rate and gradient norms indicate a controlled training environment. However, the persistent oscillations in the loss curves suggest that further optimization may be necessary to achieve smoother convergence and more stable training dynamics.

G. Extended Collision Evaluation on the COVID-19 Target with PMDM (35). We have extended our case study on the COVID-19 therapeutic target by incorporating a comprehensive comparison with PMDM, in addition to TargetDiff. All three methods are based on diffusion models, enabling a consistent evaluation of atomic-level generation dynamics. We focus on the 3CL protease, a validated COVID-19 target, and measure three types of atomic collision metrics: **PLCR**, **ALCR**, and **MLCR**, across 11 inference steps (from Step-0 to Step-1000). For fair comparison, we standardize the number of inference steps to 1000 for all models.

The new results are summarized in Table S8, Table S9, and Table S10. We observe that, while PMDM and TargetDiff both reduce collision rates over time, NucleusDiff achieves consistently lower values across all metrics and time steps. For instance, in the final inference step (Step-1000), NucleusDiff reduces **PLCR** to 3/210000, compared to 5/210000 for TargetDiff and 7267/210000 for PMDM. Similarly, NucleusDiff outperforms both baselines in **ALCR** and **MLCR**, indicating fewer atomic-level clashes during generation. These findings further validate the robustness of our proposed manifold constraint in reducing unphysical atomic collisions, and demonstrate that NucleusDiff is better aligned with physically realistic molecular generation under challenging biomedical targets.

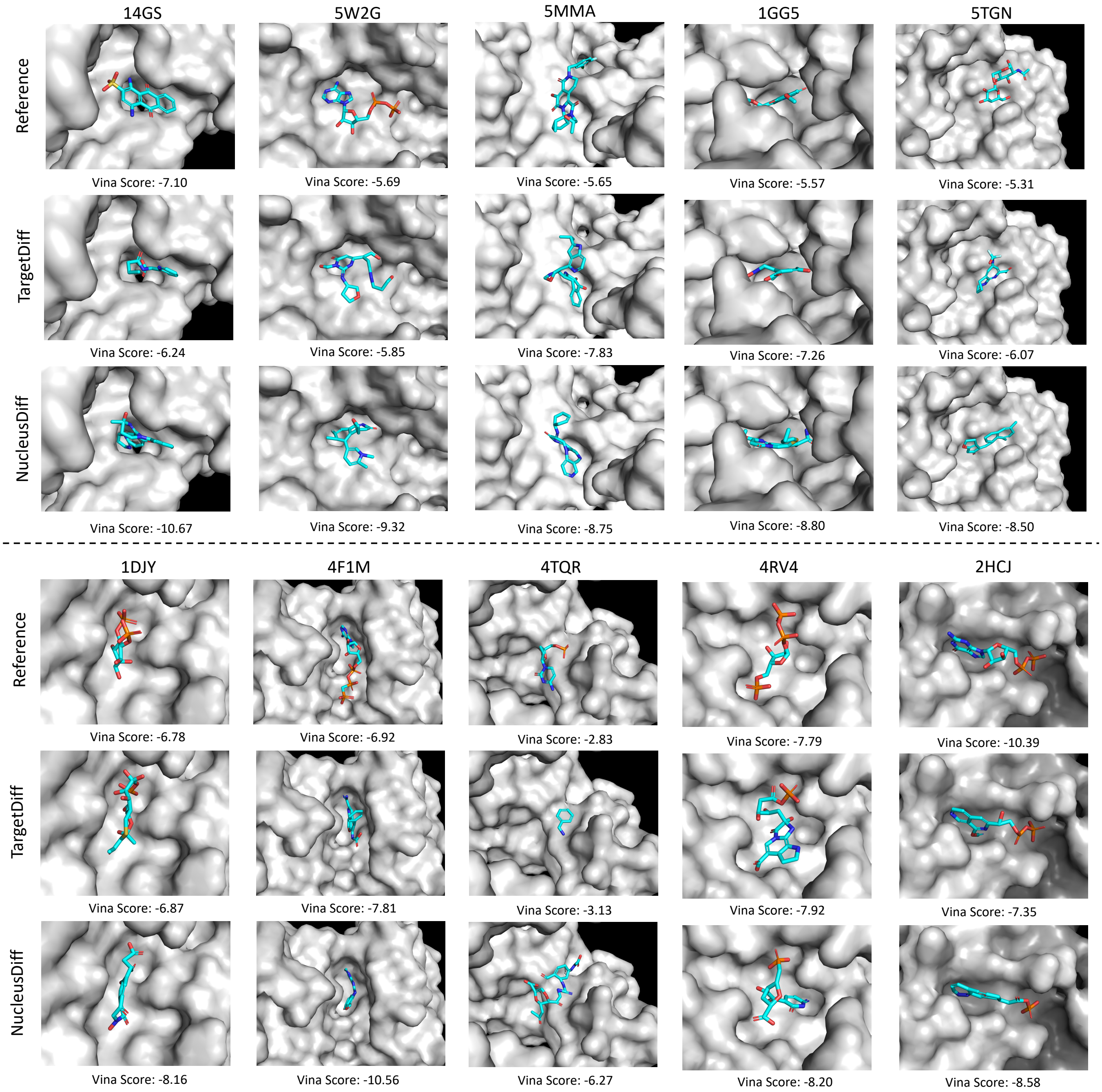

Fig. S9. More visualization of the generated molecules by TargetDiff and NucleusDiff.

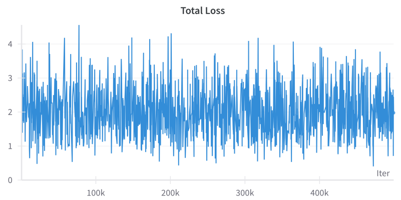

(a) Total Training Loss Curve

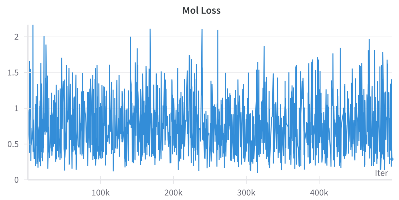

(b) Training Loss Curve for Molecular Modeling

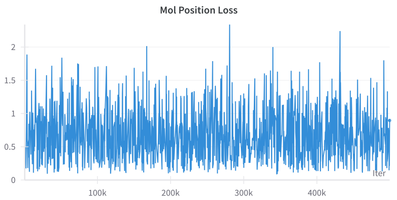

(c) Training Loss Curve for Molecular Position

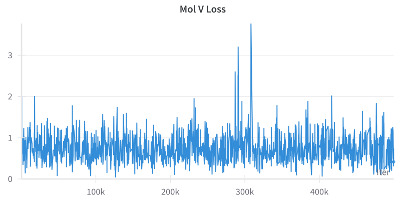

(d) Training Loss Curve for Molecular Feature

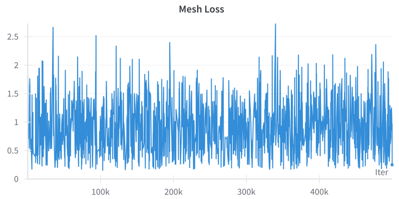

(e) Training Loss Curve for Mesh Modeling

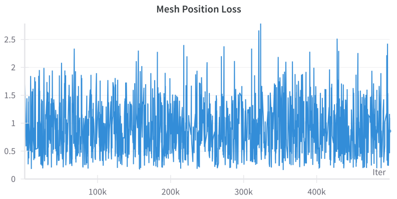

(f) Training Loss Curve for Mesh Position

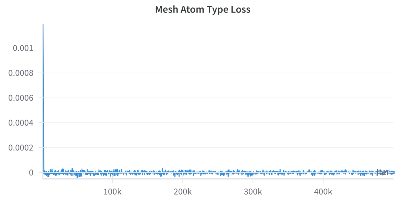

(g) Training Loss Curve for Mesh Feature

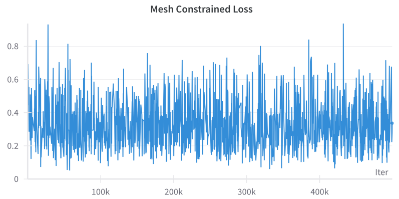

(h) Training Loss Curve for Constrained Loss

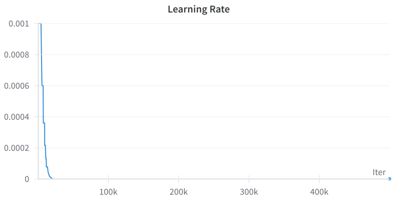

(i) Learning Rate Curve

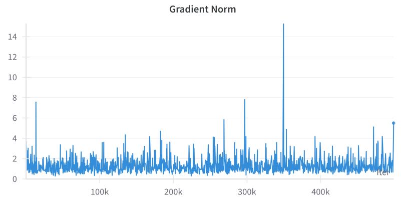

(J) Gradient Normalization Curve

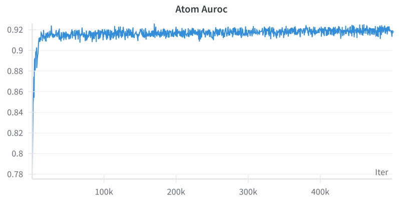

(k) Atom Type Accuracy Curve

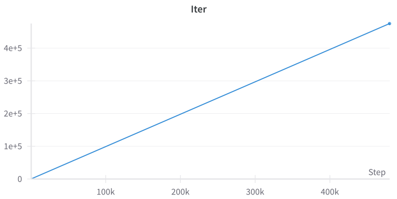

(l) Iteration Curve

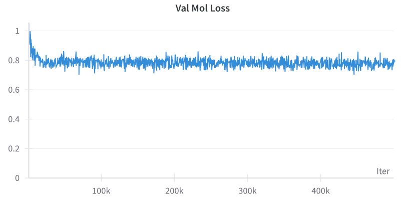

(m) Validation Loss Curve for Molecular Modeling

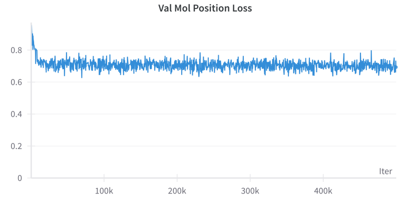

(n) Validation Loss Curve for Molecular Position

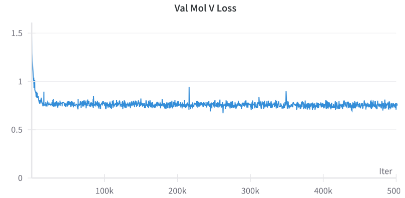

(o) Validation Loss Curve for Molecular Feature

Fig. S10. The visualization of the training process of NucleusDiff.

Table S8. The PLCR performance among pocket-ligand pairs for structure-based drug design in the COVID-19 target.

Step	PMDM	TargetDiff	NucleusDiff
**Step-0**	15945/210000	15282/210000	**15272/210000**
**Step-100**	13943/210000	9896/210000	**8940/210000**
**Step-200**	12892/210000	5388/210000	**4083/210000**
**Step-300**	12398/210000	2186/210000	**1255/210000**
**Step-400**	11703/210000	710/210000	**277/210000**
**Step-500**	11684/210000	166/210000	**51/210000**
**Step-600**	10180/210000	42/210000	**14/210000**
**Step-700**	8194/210000	23/210000	**5/210000**
**Step-800**	7427/210000	17/210000	**1/210000**
**Step-900**	7332/210000	12/210000	**1/210000**
**Step-1000**	7267/210000	5/210000	**3/210000**

Table S9. The ALCR performance among pocket-ligand pairs for structure-based drug design in the COVID-19 target.

Step	PMDM	TargetDiff	NucleusDiff
**Step-0**	10143/21000	10407/21000	**10398/21000**
**Step-100**	8880/21000	6961/21000	**6305/21000**
**Step-200**	8659/21000	3936/21000	**3047/21000**
**Step-300**	8472/21000	1630/21000	**1066/21000**
**Step-400**	8273/21000	538/21000	**233/21000**
**Step-500**	8322/21000	127/21000	**45/21000**
**Step-600**	7614/21000	36/21000	**12/21000**
**Step-700**	6448/21000	20/21000	**4/21000**
**Step-800**	5974/21000	16/21000	**1/21000**
**Step-900**	5937/21000	10/21000	**1/21000**
**Step-1000**	5869/21000	5/21000	**3/21000**

Table S10. The MLCR performance among pocket-ligand pairs for structure-based drug design in the COVID-19 target.

Step	PMDM	TargetDiff	NucleusDiff
**MLCR**	**PMDM**	**TargetDiff**	**NucleusDiff**
**Step-0**	1000/1000	1000/1000	**1000/1000**
**Step-100**	945/1000	999/1000	**999/1000**
**Step-200**	883/1000	967/1000	**958/1000**
**Step-300**	844/1000	715/1000	**634/1000**
**Step-400**	891/1000	321/1000	**204/1000**
**Step-500**	916/1000	83/1000	**40/1000**
**Step-600**	958/1000	26/1000	**11/1000**
**Step-700**	984/1000	17/1000	**4/1000**
**Step-800**	990/1000	12/1000	**1/1000**
**Step-900**	994/1000	8/1000	**1/1000**
**Step-1000**	993/1000	3/1000	**3/1000**

7. Ablation Studies

In this section, we systematically fine-tune the hyperparameters related to both the model architecture and the data processing pipeline. Additionally, we conduct comprehensive ablation studies to assess the impact of various components on overall performance. This includes a detailed sensitivity analysis of the hyperparameters to understand their influence on the model's stability and effectiveness. Our objective is to provide insights into the optimal configuration settings that enhance the model's predictive accuracy and robustness. Furthermore, we carry out rigorous experiments to evaluate the effectiveness of applying minimum distance constraints during the sampling process of the pre-trained NucleusDiff and TargetDiff models. These experiments assess the generated molecules based on both atomic collision and binding affinity performance.

A. Sensitivity Analysis of Hyperparameters in Encoder Layers. We conduct a sensitivity analysis to investigate the influence of hyperparameters in encoder layers on model stability and performance. Table S11 presents the results of varying the number of encoder layers and observing the effects on Atom Stability, Molecular Stability, and Molecular Completion Rate, alongside collision metrics such as PLCR, ALCR, and MLCR for Step-1000. The analysis reveals that increasing the number of encoder layers generally enhances model stability and completion rates. For instance, when the number of encoder layers increases from 5 to 9, Atom Stability improves from 73.75% to 74.60%, and Molecular Stability shows a notable increase from 2.48% to 3.60%. Additionally, the Molecular Completion Rate sees an improvement, rising from 85.64% to 93.60%. These results indicate that a higher number of encoder layers can positively impact the model's ability to maintain stability and achieve higher completion rates. Moreover, the PLCR and ALCR metrics remain consistent across different configurations, indicating that layer count has a minimal effect on these particular metrics. However, there is variation in MLCR, especially noticeable with 11 layers, which suggests that there might be an optimal range for the number of layers beyond which no significant gains are observed. Specifically, the configuration with 9 encoder layers shows the highest stability and completion rates, making it the optimal choice for balancing model performance and stability. Overall, this sensitivity analysis underscores the importance of fine-tuning the number of encoder layers to balance model stability and performance effectively.

Table S11. The sensitivity analysis of hyperparameters in encoder layers.

Number of Encoder Layer	Atom Stability (%)	Molecular Stability (%)	Molecular Completion Rate (%)	PLCR (Step-1000)	ALCR (Step-1000)	MLCR (Step-1000)
**5**	73.75	2.48	85.64	1/2300930	1/230093	1/10000
**6**	71.24	2.27	88.94	0/2300930	0/230093	0/10000
**7**	71.76	3.15	87.38	0/2300930	0/230093	0/10000
**8**	73.51	**4.51**	88.30	1/2300930	1/230093	1/10000
**9**	**74.60**	3.60	**93.60**	**0/2300930**	**0/230093**	**0/10000**
**10**	74.29	2.89	89.00	0/2300930	0/230093	0/10000
**11**	66.41	1.56	89.82	0/2300930	0/230093	0/10000

B. Ablation Study on Shared-Parameter Encoders for Molecular and Mesh Data. We also delve into an ablation study focusing on the use of shared-parameter encoders for both molecular and mesh data. The primary objective of this analysis is to evaluate the impact of shared-parameter backbones on key performance metrics, including Atom Stability, Molecular Stability, Molecular Completion Rate, PLCR (Step-1000), ALCR (Step-1000), and MLCR (Step-1000).

Table S12 presents the results of this ablation study, comparing configurations with and without shared-parameter backbones. It is noteworthy that employing shared-parameter backbones introduces a potential issue: the generation of molecules containing intermixed mesh points. For such mixed molecular data, we opt to discard these instances and exclude them from metric evaluation to maintain the integrity and relevance of the results.

The ablation study results indicate a significant performance degradation when using a shared-parameter backbone. Atom Stability decreases from 74.60% to 70.50%, suggesting that the inclusion of mesh data adversely impacts the stability of atomic structures, likely due to interference from mesh points within the molecular data. Molecular Stability drops significantly from 3.60% to 1.35%, indicating that the structural integrity of molecules is compromised, leading to more unstable configurations. The Molecular Completion Rate is drastically lower with the shared-parameter backbone, falling from 93.60% to 35.00%, which can be attributed to the disruptive presence of mesh points hindering the proper assembly of molecular structures. Additionally, the disruptive presence of mesh points hinders the proper assembly of molecular structures, which is evident in the collision metrics (PLCR, ALCR, MLCR for Step-1000) where no successful completions are recorded with the shared-parameter backbone, in contrast to the successful completions observed without it.

These results highlight the significant impact of shared-parameter backbones on model performance and stability. The ablation study unequivocally demonstrates that the use of shared-parameter encoders for processing both molecular and mesh data negatively impacts the stability and completeness of molecular structures. The introduction of mesh points within molecular datasets results in decreased Atom and Molecular Stability, as well as a substantially lower Molecular Completion Rate. Additionally, collision metrics suffer considerably, with no successful completions recorded when using shared-parameter backbones. Given these findings, it is evident that while shared-parameter encoders might offer computational efficiency, they compromise the modeling process of molecular data. Therefore, for applications demanding high fidelity in molecular modeling, it is advisable to avoid shared-parameter backbones or employ strategies to effectively segregate molecular and mesh data within the processing pipeline. A critical reason for avoiding the same encoder for both molecular and mesh data is that the

distribution of mesh points and molecular points is fundamentally different. This necessitates the use of distinct networks to encode these two types of data.

Table S12. The ablation study on shared-parameter encoders for molecular and mesh data.

Shared-parameter Backbone	Atom Stability (%)	Molecular Stability (%)	Molecular Completion Rate (%)	PLCR (Step-1000)	ALCR (Step-1000)	MLCR (Step-1000)
✓	70.50	1.35	35.00	0/555200	0/55520	0/10000
✗	74.60	3.60	93.60	0/2300930	0/230093	0/10000

C. Ablation Study on Mesh Point Feature Encoding Methods. We investigate the impact of different encoding strategies for mesh point features on model performance and stability. Table S13 presents the results of this ablation study, comparing the performance metrics when mesh points are encoded using the same method as atoms versus using an independent encoding type. Our analysis indicates that using an independent encoding type for mesh points yields better Atom Stability, with a value of 74.60% compared to 71.25% when using the same encoding as atoms. Furthermore, Molecular Stability also shows a slight improvement, increasing from 3.20% to 3.60%. However, the Molecular Completion Rate is higher when mesh points are encoded the same as atoms, achieving 97.84% compared to 93.60% with independent encoding. Interestingly, the collision metrics (PLCR, ALCR, MLCR) reveal a significant difference between the two encoding strategies. Employing the same encoding for both mesh points and atoms leads to a higher number of collisions among atoms and molecules, with PLCR, ALCR, and MLCR values of 3/2,300,930, 3/230,093, and 3/10,000, respectively. Conversely, the independent encoding strategy results in no recorded collisions for these metrics. The observed differences in the metrics between the two encoding strategies highlight the importance of carefully selecting the appropriate encoding method for mesh points. These results suggest that while independent encoding of mesh points may enhance stability, it may also introduce complexities that affect the overall completion rate and collision metrics. Therefore, a balance between encoding strategies should be considered to optimize both stability and completion rates in the model. The improved performance with the independent encoding type can be attributed to the model's ability to learn to distinguish between the electron cloud represented by mesh points and the atomic nuclei.

Table S13. The ablation study on mesh point feature encoding methods.

Mesh Point Feature Encoding Method	Atom Stability (%)	Molecular Stability (%)	Molecular Completion Rate (%)	PLCR (Step-1000)	ALCR (Step-1000)	MLCR (Step-1000)
**Atom-Equivalent Encoding**	71.25	3.20	97.84	3/2300930	3/230093	3/10000
**Independent Encoding Scheme**	74.60	3.60	93.60	0/2300930	0/230093	0/10000

D. The Collision Results for Minimum Distance Constraint. Our research aims to address the critical issue of atomic collisions in structure-based drug design. While NucleusDiff incorporates soft constraints during training to mitigate such collisions, an alternative strategy involves enforcing minimum distance constraints during the sampling phase of pre-trained models. In this section, we rigorously evaluate the effectiveness of applying minimum distance constraints during the sampling procedures of pre-trained NucleusDiff and TargetDiff models, focusing on the generated molecules' performance with respect to both collision metrics and binding affinity.

Given that NucleusDiff has shown near-complete elimination of atomic collision on the CrossDock2020 dataset, we extend our analysis with a more representative experiment. Specifically, we examine the properties of 1,000 molecules sampled from both the NucleusDiff and TargetDiff models using a minimum distance constraint inference process, with a particular focus on the COVID-19 target, 3CL protease. This approach allows for a more comprehensive evaluation of the models' performance under minimum distance constraint conditions, offering deeper insights into their efficacy within this context.

Minimum Distance Constraint for the Inference Process of TargetDiff and NucleusDiff. The core concept behind the minimum distance constraint is to adjust the distances between atom pairs that exhibit atomic collision during the sampling process. In this paper, we introduce two post-correction schemes based on minimum distance constraints:

Minimum Distance Constraint (Parallelogram): For a protein-ligand atom pair (a, b) exhibiting atomic collision, we first identify atom c within the ligand that is closest to ligand atom b. In 3D space, the line connecting the protein-ligand pair intersects with a sphere centered at ligand atom c, where the sphere's radius equals the distance between atoms c and b. One obvious intersection point is b, while the second intersection point, denoted as b', becomes the corrected position of atom b after applying the minimum distance constraint. This adjustment ensures a valid distance between the atoms while maintaining the ligand's geometric integrity.

Minimum Distance Constraint (Circle): For a protein-ligand atom pair (a, b) that violates collison constraints, we begin by identifying the ligand atom c closest to atom b. We ensure that the distance between atoms a and b is less than that between a and c. Based on this condition, we construct two spheres: the first is centered at protein atom a with a radius equal to the sum of the covalent radius of atoms a and b, recognizing that the distance between (a, b) is less than their combined covalent radius. The second sphere is centered at ligand atom c with a radius equal to the distance between atoms c and b. If these two spheres intersect, their intersection forms a circular region. Using an analytical expression for this circle, we sample a new position b' on the circle, guided by a predetermined random seed (42). This corrected position b' resolves the atomic collision while preserving the ligand's geometric characteristics. In cases where the two spheres are tangent, the point

of tangency serves as the unique corrected position of b', thereby avoiding atomic collisions and maintaining the structural integrity of the ligand.

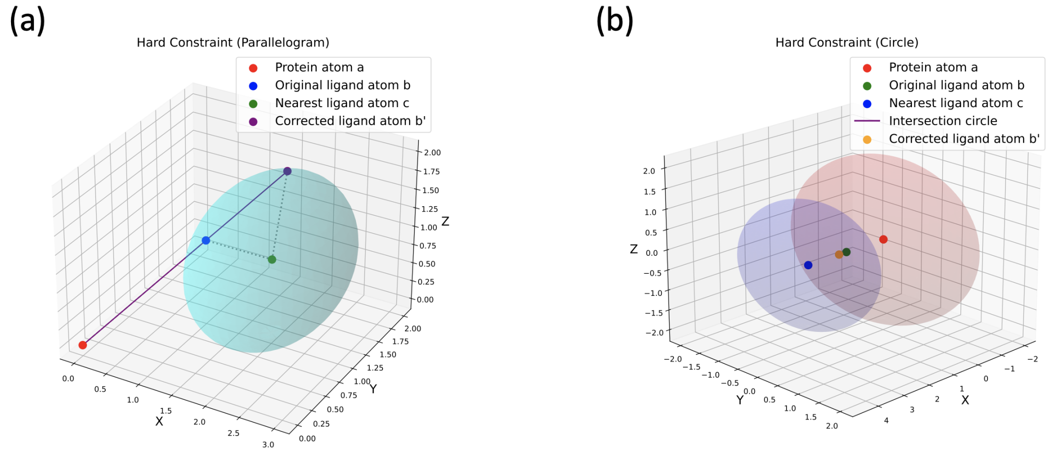

Fig. S11. The illustration of the two minimum distance constraint methods.

The Collision Evaluation of Minimum Distance Constraint for TargetDiff and NucleusDiff. The experimental results present in table S14 and table S15 demonstrate the efficacy of implementing minimum distance constraints to mitigate atomic collisions in structure-based drug design, specifically for the COVID-19 target (3CL). We evaluate the performance using three metrics: Pairwise-Level Collision Ratio (PLCR), Atom-Level Collision Ratio (ALCR), and Molecule-Level Collision Ratio (MLCR).

For TargetDiff (table S14), the baseline model without minimum distance constraints exhibits a non-negligible level of atomic collisions, with 5 collisions per 210,000 atom pairs (PLCR), 5 per 21,000 atoms (ALCR), and 3 per 1,000 molecules (MLCR). Notably, the implementation of both parallelogram and circle minimum distance constraints completely eliminates these collisions across all metrics, resulting in zero collision for all ratios.

Similarly, NucleusDiff (table S15) shows a slight improvement in the baseline performance compared to TargetDiff, with 3 collisions per 210,000 atom pairs (PLCR), 3 per 21,000 atoms (ALCR), and 3 per 1,000 molecules (MLCR). This baseline improvement can be attributed to the manifold-constrained modeling approach inherent to NucleusDiff. Nevertheless, the application of minimum distance constraints (both parallelogram and circle methods) yields the same perfect results as observed with TargetDiff, completely eliminating all atomic collisions.

These results underscore the critical importance of incorporating minimum distance constraints in the sampling process of pre-trained models for structure-based drug design. Both the parallelogram and circle constraint methods prove equally effective in resolving atomic collisions, suggesting that either approach can be reliably employed to enhance the physical realism of generated molecular structures.

The complete elimination of collisions across all metrics for both TargetDiff and NucleusDiff when using minimum distance constraints highlights the robustness of this approach. This improvement is particularly significant for the COVID-19 target (3CL), demonstrating the potential of these methods in generating more physically viable drug candidates for this crucial therapeutic target.

Table S14. TargetDiff's atomic collision performance among pocket-ligand pairs for structure-based drug design in COVID-19 target. Two types of minimum distance constraints are considered: w/ Parallelogram and w/ Circle.

Metrics	TargetDiff		
	PLCR	ALCR	MLCR
-	5/210000	5/21000	3/1000
+ Parallelogram	0/210000	0/21000	0/1000
+ Circle	0/210000	0/21000	0/1000

Table S15. NucleusDiff's atomic collision performance among pocket-ligand pairs for structure-based drug design in COVID-19 target. Two types of minimum distance constraints are considered: w/ Parallelogram and w/ Circle.

Metrics	NucleusDiff (ours)		
	PLCR	ALCR	MLCR
-	3/210000	3/21000	3/1000
+ Parallelogram	0/210000	0/21000	0/1000
+ Circle	0/210000	0/21000	0/1000

The Binding Affinity Results for Minimum Distance Constraint (TargetDiff). Here, we present a comprehensive overview of the physicochemical properties of the molecule(s) with atomic collisions generated by TargetDiff, both with and without minimum distance constraint inference in table S16, table S17 and table S18. The experimental results for TargetDiff with and without minimum distance constraints on three molecule(s) with atomic collisions (IDs: 115, 229, and 379) for the COVID-19 target 3CL reveal important insights into the trade-offs between eliminating atomic collisions and maintaining favorable physicochemical properties. For the Atomic Displacement metric, it is used to evaluate the distance that collision atoms move after correction.

For the molecule 115, the application of the parallelogram minimum distance constraint resulted in an invalid structure, suggesting that this method may sometimes lead to chemically implausible configurations. The circle minimum distance constraint, while successful in generating a valid molecule, results in generally less favorable properties. Notably, the Vina Score increased from 15.410 to 17.000, indicating reduced binding affinity. The QED (Quantitative Estimate of Drug-likeness) slightly decreased from 0.890 to 0.840, suggesting a minor reduction in overall drug-like properties. The significant atomic displacement of 3.090 Å indicates a substantial structural change, which likely contributes to the altered physicochemical properties.

For the molecule 229, in this case, the parallelogram minimum distance constraint successfully generate a valid molecule with some improvements in binding affinity. The Vina Score decreases from -0.142 to -0.712, and the Vina Dock score improves from -4.962 to -5.805, both indicating enhanced binding. Other properties remained largely unchanged, with only a minimal atomic displacement of 0.238 Å . However, the circle minimum distance constraint method fails to produce a valid molecule, highlighting the potential limitations of this approach for certain molecular structures.

For the molecule 379, both minimum distance constraint methods generate valid molecules for this case, but with some compromises in binding affinity. The Vina Score increases from 19.287 to 20.124 (parallelogram) and 20.27 (circle), indicating

slightly reduced binding affinity. The Vina Min and Vina Dock scores show mixed results, with some improvements and some deteriorations. Interestingly, other properties like QED, SA (Synthetic Accessibility), and LogP remain constant across all versions. The atomic displacements were relatively small (0.210 Å for parallelogram and 0.803 Å for circle), yet they resulted in noticeable changes in binding affinity.

Overall, these results demonstrate that while minimum distance constraints can effectively eliminate atomic collisions, they often come at the cost of altered molecular properties, particularly binding affinity. The impact varies significantly between molecules and constraint methods:

- Validity: Hard constraints can sometimes lead to invalid molecular structures, as seen with the parallelogram method for molecule 115 and the circle method for molecule 229.
- Binding Affinity: In most cases, the application of minimum distance constraints resulted in reduced binding affinity, as indicated by increased Vina Scores. However, there were exceptions, such as molecule 229 with the parallelogram constraint.
- Structural Changes: The atomic displacements varied from minimal (0.210 Å) to substantial (3.090 Å), indicating that the extent of structural modification required to resolve collisions can differ greatly between molecules.
- The effectiveness and impact of the parallelogram and circle methods varied across molecules, suggesting that the choice of minimum distance constraint method should be considered carefully for each specific case.

In conclusion, while minimum distance constraints offer a promising approach to mitigating atomic collisions in structure-based drug design, their application requires careful consideration of the potential trade-offs in molecular properties, particularly binding affinity.

Table S16. A comprehensive overview of the physicochemical properties exhibited by the molecule with atomic collisions (id:115) generated via TargetDiff for target 3CL. Two types of minimum distance constraints are considered: w/ Parallelogram and w/ Circle. The symbols (↑) and (↓) denote whether higher or lower values are deemed more favorable for each respective property.

Metrics	Vina Score (↓)	Vina Min (↓)	Vina Dock (↓)	QED (↑)	SA (↓)	LogP	Atomic Displacement (Å)
**Targetdiff**	15.410	7.470	-6.09	0.890	0.720	1.410	-
**+ Parallelogram**	Invalid	Invalid	Invalid	Invalid	Invalid	Invalid	-
**+ Circle**	17.005	6.541	-3.775	0.844	0.676	1.741	3.094

Table S17. A comprehensive overview of the physicochemical properties exhibited by the molecule with atomic collisions (id:229) generated via TargetDiff for target 3CL. Two types of minimum distance constraints are considered: w/ Parallelogram and w/ Circle. The symbols (↑) and (↓) denote whether higher or lower values are deemed more favorable for each respective property.

Metrics	Vina Score (↓)	Vina Min (↓)	Vina Dock (↓)	QED (↑)	SA (↓)	LogP	Atomic Displacement (Å)
**TargetDiff**	-0.142	-4.125	-4.962	0.251	0.630	2.110	-
**+ Parallelogram**	-0.712	-4.193	-5.805	0.250	0.630	2.110	0.238
**+ Circle**	Invalid	Invalid	Invalid	Invalid	Invalid	Invalid	-

Table S18. A comprehensive overview of the physicochemical properties exhibited by the molecule with atomic collisions (id:379) generated via TargetDiff for target 3CL. Two types of minimum distance constraints are considered: w/ Parallelogram and w/ Circle. The symbols (↑) and (↓) denote whether higher or lower values are deemed more favorable for each respective property.

Metrics	Vina Score (↓)	Vina Min (↓)	Vina Dock (↓)	QED (↑)	SA (↓)	LogP	Atomic Displacement (Å)
**Targetdiff**	19.287	-0.543	-6.393	0.467	0.610	0.478	-
**+ Parallelogram**	20.124	-0.363	-6.387	0.467	0.610	0.478	0.210
**+ Circle**	20.27	-1.827	-6.075	0.467	0.610	0.478	0.803

The Binding Affinity Results for Minimum Distance Constraint (NucleusDiff). A comprehensive overview of the physicochemical properties exhibited by the collision molecules generated via NucleusDiff is presented in table S19, table S20, and table S21. For molecule ID 50, all three methods (NucleusDiff, + Minimum Distance Constraint (Parallelogram), and + Minimum Distance Constraint (Circle)) result in invalid structures, indicating that the minimum distance constraint methods are unable to generate valid molecules for this particular case.

In contrast, molecule 135 yields more promising results. The baseline NucleusDiff method produces a valid molecule with a Vina Score of -5.946, indicating a relatively strong binding affinity. However, the application of the parallelogram minimum distance constraint results in an invalid structure, suggesting that this method may not be suitable for this particular molecule.

On the other hand, the circle minimum distance constraint method generates a valid molecule with a Vina Score of -5.939, which is comparable to the baseline result. Additionally, the atomic displacement of 1.024 Å indicates a relatively small structural change, which may be beneficial for preserving the molecular properties.

Unfortunately, molecule 353 exhibits similar results to molecule 50, with all three methods resulting in invalid structures. This suggests that the minimum distance constraint methods may not be effective for this particular molecule, and alternative approaches may be needed to generate valid structures with improved binding affinity.

Overall, the results suggest that the effectiveness of the minimum distance constraint methods for NucleusDiff is highly dependent on the specific molecule being studied. While some molecules may benefit from the application of minimum distance constraints, others may result in invalid structures or reduced binding affinity. Further research is needed to develop more robust and generalizable methods for improving the binding affinity of molecules generated by NucleusDiff.

Table S19. A comprehensive overview of the physicochemical properties exhibited by the molecule with atomic collisions (id:50) generated via NucleusDiff for target 3CL. Two types of minimum distance constraints are considered: w/ Parallelogram and w/ Circle. The symbols (↑) and (↓) denote whether higher or lower values are deemed more favorable for each respective property.

Metrics	Vina Score (↓)	Vina Min (↓)	Vina Dock (↓)	QED (↑)	SA (↓)	LogP	Atomic Displacement (Å)
**NucleusDiff (ours)**	Invalid	Invalid	Invalid	Invalid	Invalid	Invalid	-
**+ Parallelogra**	Invalid	Invalid	Invalid	Invalid	Invalid	Invalid	-
**+ Circle**	Invalid	Invalid	Invalid	Invalid	Invalid	Invalid	-

Table S20. A comprehensive overview of the physicochemical properties exhibited by the molecule with atomic collisions (id:135) generated via NucleusDiff for target 3CL. Two types of minimum distance constraints are considered: w/ Parallelogram and w/ Circle. The symbols (↑) and (↓) denote whether higher or lower values are deemed more favorable for each respective property.

Metrics	Vina Score (↓)	Vina Min (↓)	Vina Dock (↓)	QED (↑)	SA (↓)	LogP	Atomic Displacement (Å)
**NucleusDiff (ours)**	-5.946	-7.055	-7.646	0.308	0.360	1.790	-
**Parallelogram**	Invalid	Invalid	Invalid	Invalid	Invalid	Invalid	-
**Circle**	-5.939	-6.516	-6.441	0.308	0.360	1.790	1.024

Table S21. A comprehensive overview of the physicochemical properties exhibited by the molecule with atomic collisions (id:353) generated via NucleusDiff for target 3CL. Two types of minimum distance constraints are considered: w/ Parallelogram and w/ Circle. The symbols (↑) and (↓) denote whether higher or lower values are deemed more favorable for each respective property.

Metrics	Vina Score (↓)	Vina Min (↓)	Vina Dock (↓)	QED (↑)	SA (↓)	LogP	Atomic Displacement (Å)
**NucleusDiff (ours)**	Invalid	Invalid	Invalid	Invalid	Invalid	Invalid	-
**+ Parallelogram**	Invalid	Invalid	Invalid	Invalid	Invalid	Invalid	-
**+ Circle**	Invalid	Invalid	Invalid	Invalid	Invalid	Invalid	-

Movie S1. A demo illustrating the inference process of the TargetDiff and NucleusDiff.

References

1. M Skalic, D Sabbadin, B Sattarov, S Sciabola, G De Fabritiis, From target to drug: generative modeling for the multimodal structure-based ligand design. *Mol. pharmaceutics* **16**, 4282–4291 (2019).
2. M Xu, T Ran, H Chen, De novo molecule design through the molecular generative model conditioned by 3d information of protein binding sites. *J. Chem. Inf. Model.* **61**, 3240–3254 (2021).
3. C Tan, Z Gao, SZ Li, Target-aware molecular graph generation. *arXiv preprint arXiv:2202.04829* (2022).
4. M Ragoza, T Masuda, DR Koes, Generating 3D molecules conditional on receptor binding sites with deep generative models. *Chem Sci* **13**, 2701–2713 (2022).
5. F Sun, Z Zhan, H Guo, M Zhang, J Tang, Graphvf: Controllable protein-specific 3d molecule generation with variational flow. (2023).
6. Y Li, J Pei, L Lai, Structure-based de novo drug design using 3d deep generative models. *Chem. science* **12**, 13664–13675 (2021).
7. S Luo, J Guan, J Ma, J Peng, A 3d generative model for structure-based drug design. *Adv. Neural Inf. Process. Syst.* **34** (2021).
8. M Liu, Y Luo, K Uchino, K Maruhashi, S Ji, Generating 3d molecules for target protein binding in *International Conference on Machine Learning.* (2022).
9. X Peng, et al., Pocket2mol: Efficient molecular sampling based on 3d protein pockets. *arXiv preprint arXiv:2205.07249* (2022).
10. T Lin, H Zha, Riemannian manifold learning. *IEEE transactions on pattern analysis machine intelligence* **30**, 796–809 (2008).
11. X He, P Niyogi, Locality preserving projections. *Adv. neural information processing systems* **16** (2003).
12. JB Tenenbaum, Vd Silva, JC Langford, A global geometric framework for nonlinear dimensionality reduction. *science* **290**, 2319–2323 (2000).
13. ST Roweis, LK Saul, Nonlinear dimensionality reduction by locally linear embedding. *science* **290**, 2323–2326 (2000).
14. W Liu, et al., Sphereface: Deep hypersphere embedding for face recognition in *Proceedings of the IEEE conference on computer vision and pattern recognition.* pp. 212–220 (2017).
15. D Boscaini, M Bronstein, B Correia, Deciphering interaction fingerprints from protein molecular surfaces using geometric deep learning. *Nat. Methods* **17**, 184–192 (2020).
16. O Zhang, et al., Learning on topological surface and geometric structure for 3d molecular generation. *Nat. Comput. Sci.* pp. 1–11 (2023).
17. Y Wang, et al., Learning harmonic molecular representations on riemannian manifold in *The Eleventh International Conference on Learning Representations.* (2022).
18. V Mallet, S Attaiki, M Ovsjanikov, Atomsurf: Surface representation for learning on protein structures. *arXiv preprint arXiv:2309.16519* (2023).
19. VG Satorras, E Hoogeboom, M Welling, E (n) equivariant graph neural networks in *International Conference on Machine Learning.* (PMLR), (2021).
20. S Liu, et al., Symmetry-informed geometric representation for molecules, proteins, and crystalline materials. *arXiv preprint arXiv:2306.09375* (2023).
21. W Du, J Chen, X Zhang, Z Ma, S Liu, Molecule joint auto-encoding: Trajectory pretraining with 2d and 3d diffusion. *arXiv preprint arXiv:2312.03475* (2023).
22. S Liu, W Du, ZM Ma, H Guo, J Tang, A group symmetric stochastic differential equation model for molecule multi-modal pretraining in *International Conference on Machine Learning.* (PMLR), pp. 21497–21526 (2023).
23. S Liu, H Guo, J Tang, Molecular geometry pretraining with se (3)-invariant denoising distance matching. *arXiv preprint arXiv:2206.13602* (2022).
24. VG Satorras, E Hoogeboom, M Welling, E (n) equivariant graph neural networks in *International Conference on Machine Learning.* (PMLR), pp. 9323–9332 (2021).
25. PG Francoeur, et al., Three-dimensional convolutional neural networks and a cross-docked data set for structure-based drug design. *J. Chem. Inf. Model.* **60**, 4200–4215 (2020).
26. Y Luo, S Ji, An autoregressive flow model for 3d molecular geometry generation from scratch in *International Conference on Learning Representations (ICLR).* (2022).
27. J Guan, et al., 3d equivariant diffusion for target-aware molecule generation and affinity prediction in *The Eleventh International Conference on Learning Representations.* (2022).
28. M Steinegger, J Söding, Mmseqs2 enables sensitive protein sequence searching for the analysis of massive data sets. *Nat. biotechnology* **35**, 1026–1028 (2017).
29. G Ewing, J Hermisson, Msms: a coalescent simulation program including recombination, demographic structure and selection at a single locus. *Bioinformatics* **26**, 2064–2065 (2010).
30. Q Zhou, Pymesh—geometry processing library for python (2019).
31. DP Kingma, J Ba, Adam: A method for stochastic optimization. *arXiv preprint arXiv:1412.6980* (2014).

32. A Vaswani, et al., Attention is all you need. *Adv. neural information processing systems* **30** (2017).
33. AQ Nichol, P Dhariwal, Improved denoising diffusion probabilistic models in *International Conference on Machine Learning.* (PMLR), pp. 8162–8171 (2021).
34. J Ho, A Jain, P Abbeel, Denoising diffusion probabilistic models. *Adv. neural information processing systems* **33**, 6840–6851 (2020).
35. L Huang, et al., A dual diffusion model enables 3d molecule generation and lead optimization based on target pockets. *Nat. Commun.* **15**, 2657 (2024).
